# The Value of Social Media Analysis for Adverse Events Detection and Pharmacovigilance: Scoping Review

**DOI:** 10.2196/59167

**Published:** 2024-09-06

**Authors:** Su Golder, Karen O'Connor, Yunwen Wang, Ari Klein, Graciela Gonzalez Hernandez

**Affiliations:** 1 University of York York United Kingdom; 2 University of Pennsylvannia Philadelphia, PA United States; 3 Cedars-Sinai Medical Center Los Angeles, CA United States

**Keywords:** adverse events, pharmacovigilance, social media, real-world data, scoping review

## Abstract

**Background:**

Adverse drug events pose an enormous public health burden, leading to hospitalization, disability, and death. Even the adverse events (AEs) categorized as nonserious can severely impact on patient’s quality of life, adherence, and persistence. Monitoring medication safety is challenging. Web-based patient reports on social media may be a useful supplementary source of real-world data. Despite the growth of sophisticated techniques for identifying AEs using social media data, a consensus has not been reached as to the value of social media in relation to more traditional data sources.

**Objective:**

This study aims to evaluate and characterize the utility of social media analysis in adverse drug event detection and pharmacovigilance as compared with other data sources (such as spontaneous reporting systems and the clinical literature).

**Methods:**

In this scoping review, we searched 11 bibliographical databases and Google Scholar, followed by handsearching and forward and backward citation searching. Each record was screened by 2 independent reviewers at both the title and abstract stage and the full-text screening stage. Studies were included if they used any type of social media (such as Twitter or patient forums) to detect AEs associated with any drug medication and compared the results ascertained from social media to any other data source. Study information was collated using a piloted data extraction sheet. Data were extracted on the AEs and drugs searched for and included; the methods used (such as machine learning); social media data source; volume of data analyzed; limitations of the methodology; availability of data and code; comparison data source and comparison methods; results, including the volume of AEs, and how the AEs found compared with other data sources in their seriousness, frequencies, and expectedness or novelty (new vs known knowledge); and conclusions.

**Results:**

Of the 6538 unique records screened, 73 publications representing 60 studies with a wide variety of extraction methods met our inclusion criteria. The most common social media platforms used were Twitter and online health forums. The most common comparator data source was spontaneous reporting systems, although other comparisons were also made, such as with scientific literature and product labels. Although similar patterns of AE reporting tended to be identified, the frequencies were lower in social media. Social media data were found to be useful in identifying new or unexpected AEs and in identifying AEs in a timelier manner.

**Conclusions:**

There is a large body of research comparing AEs from social media to other sources. Most studies advocate the use of social media as an adjunct to traditional data sources. Some studies also indicate the value of social media in understanding patient perspectives such as the impact of AEs, which could be better explored.

**International Registered Report Identifier (IRRID):**

RR2-10.2196/47068

## Introduction

### Background

Adverse drug events (ADEs) can lead to increased morbidity, mortality, and economic burden within the health care system [1,2]. Moreover, ADEs can result in patients prematurely discontinuing treatment or being hesitant to initiate drug therapies, depriving them of potentially beneficial treatment [3]. Despite efforts to detect ADEs before a drug is marketed, some may go undetected, underscoring the importance of continuous safety surveillance and monitoring.

Postmarketing pharmacovigilance relies on spontaneous reporting to regulatory agencies, but such systems have limitations, including time delays and underreporting [4-7]. The insufficient rate of reporting has prompted researchers to explore alternative data sources.

Social media data analysis has been applied in various health research areas, such as disease surveillance and health outcomes research [8-10]. Safety outcomes, in particular, have been extensively studied [8-10], and patient reports of ADEs are found abundantly within this content-rich resource [11]. The use of social media as a supplementary data source may hold immense value, as it can capture the perspectives of patients from diverse demographics, including those who are typically not reached in traditional pharmacovigilance channels. The synthesis of ADEs reported in different data sources, including social media, may increase the representativeness and comprehensiveness of drug safety signals.

The potential value of extracting drug safety data from social media was recognized as early as 2010 [11]. Social media data were believed to have the potential to identify new signals or detect signals earlier than conventional methods [12]. To manage the vast amounts of text-based information posted on social media, ongoing advancements in natural language processing (NLP) and machine learning methods have facilitated automatic detection of relevant mentions [13,14]. These methods face numerous challenges, such as the highly informal language used on social media and extracting user–expressed ADE concepts, which are usually descriptive and nontechnical [15,16]. NLP has played a crucial role in overcoming some of these barriers encountered in identifying ADE mentions [13,14]. While technological methods continue to advance [17-21], the practical utility of social media for identifying adverse events (AEs) requires further demonstration [22], leading to an ongoing debate regarding what social media can bring to pharmacovigilance.

Numerous studies have concluded that social media holds the potential to improve pharmacovigilance, while others, including the well-known WEB-RADR study [23], have argued against it, stating that signal detection in Twitter and Facebook “performs poorly and cannot be recommended at the expense of other pharmacovigilance activities” [24]. However, these studies often make conclusions based on case studies, which necessarily present a limited perspective, particularly given the selection and the comparative analysis methods used for their case study may have impacted the outcomes. The general question of whether social media can enhance pharmacovigilance may be more complex and nuanced than a simple “yes” or “no” answer. Instead, we propose to focus this study on establishing how social media data can contribute to pharmacovigilance.

Between 2015 and 2021, 7 systematic reviews were published aiming to evaluate the potential use of social media in pharmacovigilance [25-30]. These reviews focused on various aspects such as the frequency of AE reports or the detection of safety signals [25-30]. Despite the inclusion of a substantial number of articles, these reviews generally concluded that the research was still in its infancy and that further investigations were required. Nonetheless, some of the reviews did note that social media may be more suitable for identifying mild symptomatic ADEs, gaining patient perspectives of notable events and their impact, or detecting AE signals earlier than regulatory agencies. Since the publication of these reviews, there has been significant progress in methods used to extract data from social media and numerous additional studies.

### Objective

Given the breadth of original studies conducted since these systematic reviews were published, our aim was to provide an updated summary of the current literature regarding the value of detecting ADEs from social media data as compared with other (traditional) sources. Thus, we narrowed our review to studies that included a comparison of ADEs found in social media to another (traditional) data source and excluded studies primarily focused on the technical aspects of extracting ADE reports. Considering the extensive landscape of literature in this area and our objective to map the evidence comprehensively, we chose to conduct a scoping review using the framework developed by Arksey and O’Malley [31]. Specifically, our review aimed to address the following questions:

1. What recent (post-2017) research has been conducted on the large-scale detection of AEs from social media?
2. What types of drugs and AEs have been studied using social media data to date, and what are the findings?
3. How do the types and frequency of ADEs identified from social media differ from those identified from other sources (such as regulatory data or clinical trials)?
4. What methods are used to identify and extract ADEs from social media data, and could the choice of methods impact the results?

## Methods

### Overview

This scoping review is reported in line with PRISMA-ScR (Preferred Reporting Items for Systematic Reviews and Meta-Analyses Extension for Scoping Reviews) checklist [32] and followed a prespecified published protocol [33]. The inclusion and exclusion criteria are listed in Textbox 1. The inclusion criteria were necessarily broad in nature to provide an understanding of the volume and diversity of the research in this area.

**Inclusion and exclusion criteria for studies on identifying adverse drug events data from social media in comparison with other data sources.**

**Inclusion criteria**

- Population
  Any person (including pregnant persons and young and older adults) with or without any condition or disease type (chronic or acute) who states that they have taken any drug intervention (including vaccines) used in diagnosis, treatment or prevention (as defined by the Food and Drug Administration [FDA]) and experienced an adverse event
- Intervention
  Any type of social media, defined as any computer-mediated tools for users to create, share or exchange information, ideas, or content via text, images, and audio (eg, message postings, pictures, and videos) in virtual communities and networks (such as message boards, social networks, patient forums, Twitter, Reddit, blogs, and Facebook)
- Comparator
  Any data source other than social media (such as spontaneous reporting systems of the FDA or Medicines and Healthcare products Regulatory Agency, clinical trials or summary of product characteristics) is eligible as a comparator (Table S1 in Multimedia Appendix 1)
- Outcome
  Primary outcomes: data on the type and frequency of adverse drug events data (such as muscle ache, headache, or rash) are required from social media and at least 1 other data source
  Secondary outcomes: data on the application of the adverse drug events data (such as pharmacovigilance and hypothesis generation)
- Study design
  Any type of assessment
- Any date or language limits
  Published 2017 onward in English, Spanish, or French, or in any language with an English translation available

**Exclusion criteria**

- Population
  Reports by health care professionals
  People reporting diagnosis, treatment, or prevention with a nonmedical intervention (such as medical devise, surgery, supplements, or natural remedy)
  People not reporting experience of an adverse event
- Intervention
  Simple, nonsocial, internet-based interventions (ie, web 1.0)
  Studies using social media to recruit participants
- Comparator
  No comparison undertaken to any nonsocial media data source
- Outcome
  We are concerned with the properties of interventions under normal use. We, therefore, did not consider papers where the primary aim was to assess events, such as intentional and accidental poisoning (ie, overdose), drug abuse, errors, or noncompliance. Drug-drug interactions are not eligible if they are the primary objective of the paper, due to the different techniques required in identifying interactions as opposed to adverse events under normal use.
  Papers focused on identifying patient’s perspectives of adverse events (such as fear or impact on quality of life) and papers on subsequent patient behaviors as a result of adverse events are also ineligible.
- Study design
  Discussion papers, purely technical papers, and papers that only contain examples of posts from social media.
- Any date or language limits
  Anything published before 2017 and anything published since 2017 that is not in either English, Spanish, or French, or in another language with no available English translation

### Search Methods

Eleven databases covering a range of topic areas, including health and medical research, nursing, information and computer science, and gray literature were searched (Textbox 2 and Table S2 in Multimedia Appendix 1). We also searched Google Scholar. However, due to the immense number of hits this search engine retrieves, we only sifted the first 300 records. Searching in databases may not retrieve all relevant available studies as there are delays in indexing, they may not have been indexed adequately (particularly where the database does not index using full text or uses automated methods), or they may lack detail in their titles and abstracts. We, therefore, conducted handsearching of the most common journal titles from a previous review [25]: *Drug Safety*, *Journal of Medical Internet Research*, and *Pharmacoepidemiology and Drug Safety* (2017-2023l; Textbox 2).

**Sources searched for included studies.**

**Databases**

- ACM Digital Library
- Conference Proceedings Citation Index–Science (CPCI-S)
- Emerging Sources Citation Index (ESCI)
- Embase
- IEEE Xplore
- Library, Information Science & Technology Abstracts (LISTA)
- MEDLINE
- Open Dissertations
- ProQuest dissertations and theses: United Kingdom and Ireland
- PsycINFO
- Science Citation Index Expanded (SCI-Expanded)

**Internet search engine**

- Google Scholar (first 300 records sifted)
- Handsearching of journals:
- *Drug Safety* (2017-2023)
- *Journal of Medical Internet Research* (2017-2023)
- *Pharmacoepidemiology and Drug Safety* (2017-2023)

The database search strategies consisted of just 2 facets, “social media” and “adverse events” (see Multimedia Appendix 1 for full search strategies in all databases). A date restriction of 2017 onward was placed on the searches because this review updates 7 previous reviews [25-30], the most recent of which is more focused than our review [29]. No language restrictions were placed on the searches, although financial and logistical restraints did not allow translation from all languages.

We also conducted forward and backward citation searching by checking the references of all included studies and forward citation searching using CitationChaser [34] to identify papers that have cited our included studies or that was cited by our included studies (Table S3 in Multimedia Appendix 1). We noted any related systematic reviews during our full-text screening stage and carried out forward citation searches on these reviews.

The search results were entered into an EndNote (Clarivate) library with the duplicates removed. Title and abstract screening were undertaken independently by 2 reviewers in Covidence (Covidence AS) with any disagreements resolved by discussion, or if necessary, a third reviewer. Full-text screening was again undertaken in Covidence by 2 independent reviewers.

### Data Extraction

A data extraction spreadsheet was designed and piloted for this review in Covidence. The form recorded study characteristics of existing papers on using social media data to identify potential ADEs. Two reviewers (SG and KO) extracted descriptive data independently, with findings compared and agreed through discussion and consensus with a third person where required. The following data were extracted from the included studies:

1. Details on the type of social media platform used
2. Details on the primary aim of the study
3. Brief details of the methods used to extract data from social media including which drugs or AEs are searched for and how
4. Whether the study distinguished between personal and nonpersonal mentions, and whether it accounted for the influence of bots or nonindividual accounts
5. The type and frequency of AEs data identified for each drug and which drug
6. Comparator data source or sources along with any comparisons of the data collected
7. Conclusions of the original investigators
8. Finally, whether code or annotated or raw data are made available by the authors

As this is a scoping review, we did not assess the methodological quality (risk of bias assessment) of the studies or conduct any evidence synthesis. Nevertheless, we did briefly summarize whether the methods were reported, and any issues raised.

### Ethical Considerations

Because the scoping review methodology consists of reviewing and collecting data from publicly accessible materials, this study did not require any ethical approval.

## Results

### Overview

**Figure 1:**
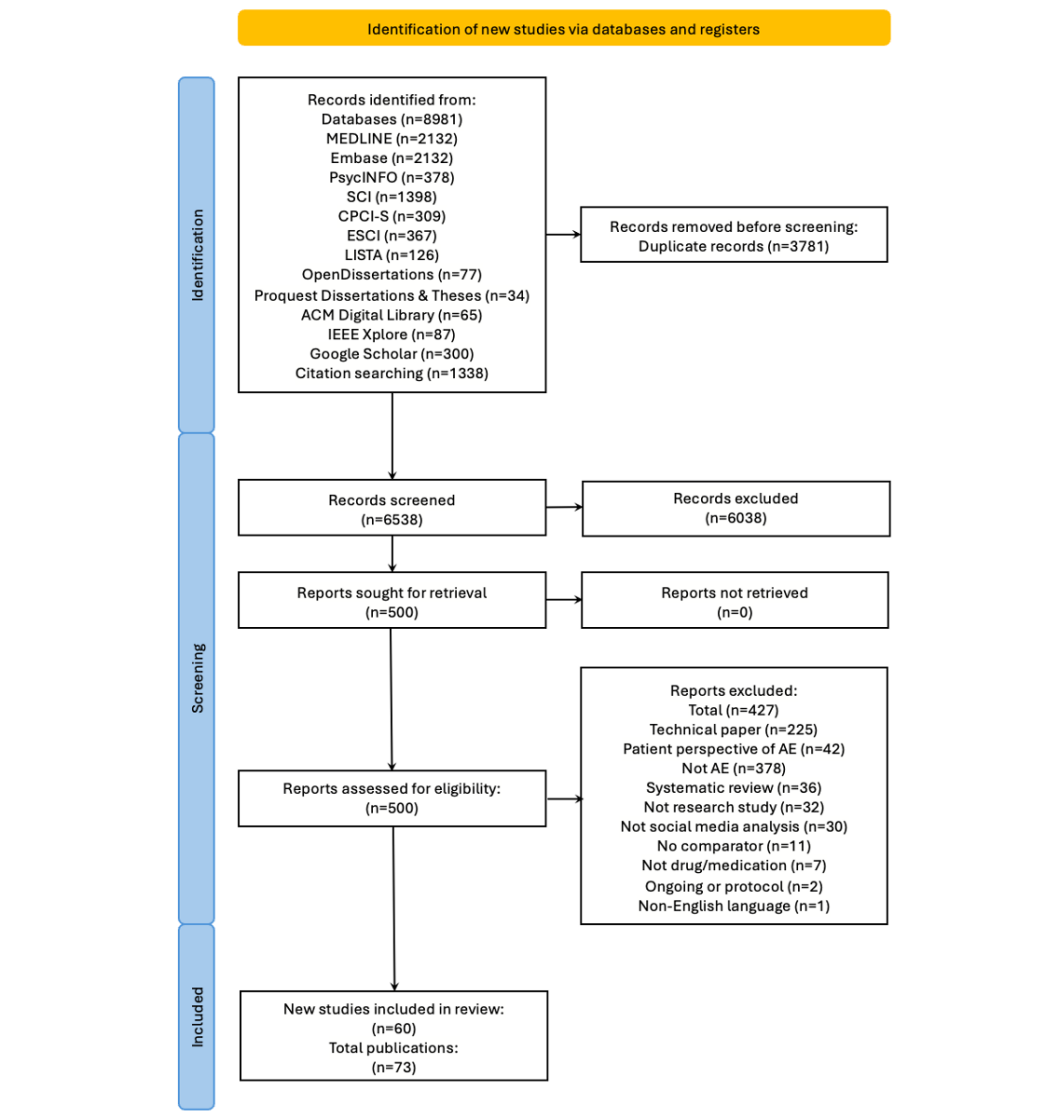
Flow diagram for included studies.

After screening 6538 unique records, the full text of 500 were examined and 73 publications representing 60 studies were included in this review (Figure 1 and Table S4 in Multimedia Appendix 1). Those excluded at the full-text stage fell into 10 categories: technical papers (n=225), patient perspective of AE (n=42), not AEs (n=41), systematic review (n=36), not research study (n=32), not social media analysis (n=30), no comparator (n=11), not drug medication (n=7), ongoing or protocol (n=2), and non-English language (Portuguese).

A brief overview of the included studies can be found in Table 1. The full details of the extracted information for each publication are provided in Table S4 in Multimedia Appendix 1.

**Table 1:** Overview of included publications and studies and their findings when comparing the adverse event extracted from social media to other data sources.

Publication (author, year)	Study name or identifier used	Social media source used	Reported finding on adverse events found in social media^a^
Abbasi et al [35], 2019	—^b^	Twitter, health forums, and drug review sites	Unexpected, earlier
Audeh et al [36], 2020	Vigi4Med	Twitter, health forums, and drug review site	Less serious, unexpected
Bellet et al [37], 2018	Vigi4Med	Twitter, health forums, and drug review site	Less serious, unexpected
Boeuf et al [38], 2017	Vigi4Med	Twitter, health forums, and drug review site	Less serious, unexpected, less informative
Karapetiantz et al [39], 2018	Vigi4Med	Twitter, health forums, and drug review site	Less serious, unexpected
Karapetiantz et al [40], 2018	Vigi4Med	Twitter, health forums, and drug review site	Less serious, unexpected
Karapetiantz et al [41], 2019	Vigi4Med	Twitter, health forums, and drug review site	Less serious
Karapetiantz et al [42], 2019	Vigi4Med	Twitter, health forums, and drug review site	Less serious, unexpected
Barakat and ElSabbagh [43], 2022	—	Health forums	New, similar, more frequent
Bennett et al [44], 2022	—	Twitter	Not reported
Bhattacharya et al [45], 2017	—	Twitter, Reddit, and health forums	Less serious, similar, less frequent
Blaser et al [46], 2017	—	Health forums	Less frequent
Borchert et al [47], 2019	—	Drug review site	Similar
Brattig [48], 2019	—	Twitter and Instagram	Similar
Campillos-llanos et al [49], 2019	—	Health forums	New
Caster et al [24], 2018	WEB-RADR	Twitter, Facebook, and health forums	Less frequent, no value
van Stekelenborg et al [50], 2019	WEB-RADR	Twitter, Facebook, and health forums	Not earlier, no value
Chen et al [51], 2018	—	Health forums	New, similar
de Langen et al [52], 2017	—	Twitter, health forums	Less serious, different pattern
den Hollander et al [53], 2022	den Hollander 2022	Facebook	Similar
Dirkson et al [54], 2022	den Hollander 2022	Facebook	New
de Rosa et al [55], 2021	—	Twitter	Similar
Dreyfus and Pierce [56], 2017	—	Twitter, Facebook, blogs, and health forums	Similar
Eslami et al [57], 2020	—	Health forums	New, less frequent
Farooq et al [58], 2020	—	Twitter	Underreported
Ferawati et al [59], 2022	—	Twitter	Less frequent
Gavrielov-Yusim et al [60], 2019	—	Health forums	Earlier, new, similar
Golder et al [61], 2021	—	Twitter	Less serious, similar
Han et al [62], 2020	—	Drug review site	Similar, less frequent
Harpster and Hultgren [63], 2018	—	Twitter	Less frequent
Hoang et al [64], 2018	—	Twitter	New, similar
Hussain et al [65], 2022	—	Twitter and Facebook	Similar
Jarynowski et al [66], 2021	—	Health forums	Similar
Jiang et al [67], 2020	—	Twitter	New, unexpected, similar
Khademi Habibabadi et al [68], 2023	—	Twitter	Similar
Kim et al [69], 2020	—	Drug review site	Similar
Koutkias et al [70], 2017	—	Twitter	Similar
Kurzinger et al [71], 2018	Kurzinger AB	Health forums	Earlier
Kurzinger et al [72], 2018	Kurzinger AB	Health forums	Earlier, new
Lardon et al [73], 2018	—	Twitter	Less serious, unexpected
Lebanova et al [74], 2019	—	Health forums	Similar
Lee et al [75], 2023	—	Naver	Similar
Li et al [76], 2019	—	Health forums	Similar
Li et al [77], 2020	—	Twitter	Similar, less frequent, less serious
Lian et al [78], 2022	—	Twitter	Similar, less serious
Liu [79], 2017	—	Twitter and health forums	Earlier, more frequent, less serious
Mackinlay et al [80], 2017	—	Twitter	New, less serious
Maskell [81], 2017	—	Twitter and Facebook	Different patterns
Matsuda et al [82], 2017	Matsuda AB	Health forums	Similar, less serious
Matsuda et al [83], 2017	Matsuda AB	Health forums	Similar, less serious
Natsiavas et al [84], 2017	—	Twitter	New
Nguyen et al [85], 2017	—	Twitter, Reddit, and blogs	Similar
Nikfarjam et al [86], 2019	Nikfarjam and Ransohoff	Health forums	Earlier, similar
Ransohoff et al [87], 2018	Nikfarjam and Ransohoff	Health forums	Earlier, new, similar
Ransohoff et al [88], 2018	Nikfarjam and Ransohoff	Health forums	Earlier, new
Oyebode and Orji [21], 2023	—	Health forums	Similar
Pan et al [89], 2018	—	Health forums	New, similar, less frequent
Park et al [90], 2022	—	Drug review site	New, unexpected
Patel et al [91], 2018	—	Twitter	Less serious
Pathak and Catalan-Matamoros [92], 2023	—	Twitter	Earlier, new, similar
Pierce et al [93], 2017	—	Twitter and Facebook	Earlier
Powell et al [94], 2022	—	Twitter and health forums	Similar, less frequent
Rees et al [95], 2018	—	Twitter and health forums	Less serious
Sadeghi et al [96], 2017	—	Health forums	Less serious
Salamun et al [97], 2020	—	Reddit	Other
Sampathkumar [98], 2017	—	Health forums and drug review site	Earlier, new, similar
Smith et al [99], 2018	—	Twitter	Similar, different rates
Song et al [100], 2021	—	Drug review site	Similar
Xia [101], 2022	—	Drug review site	Earlier, new
Yahya and Asiri [102], 2022	Yahya AB	Health forums and drug review site	Similar, less frequent
Yahya et al [103], 2022	Yahya AB	Health forums and drug review site	Similar, less frequent
Yu and Vydiswaran [104], 2022	—	Twitter	New, similar
Zhou and Hultgren [105], 2020	—	Twitter	New, similar

^a^As compared with comparator source used.

^b^Not available.

### Characteristics of Included Studies

The most commonly used social media platform was Twitter (34/60, 57%) [24,35-42,44,45,48,50,52,55,56,58,59,61, 63-65,67,68,70,73,77-81,84,85,91-95,99,104,105], followed by various health forums (26/60, 43%) [21,24,35-43,45,46,49-52,56,57,60,69,71,72,74,76,79,82,83,85-89,94-96,98,102,103], drug reviews sites (9/60, 15%) [21,35,47,62,90,98,100-103], Facebook (6/60 10%) [36-38,41,42,53,54,56,65,81], Reddit (3/60 5%) [45,85,97], blogs (3/60, 5%) [56,75,85], and other social media platforms (2/60, 3%) such as Telegram [66] and Instagram [48]. Table 2 provides an overview of these characteristics, along with references, as well as those for the remainder of this section. In studies that reported the number of drugs included, the range varied from 1 to 4888, with some studies searching for any or all named drugs within the corpus, and in many cases, not all drugs were explicitly named. This made any detailed analysis by type of drug too challenging. Furthermore, 55% (33/60) of the studies searched for data for ≤10 named drugs, 23% (14/60) of the studies searched for 11 to 200 named drugs, and 12% (7/60) of the studies searched for or extracted all named drugs in their collected corpus. Five studies did not report the exact number of drugs searched or extracted [52,81-83,90,96]. One study searched for posts of interest using 4 named AEs and then extracted drugs mentioned in these posts. Most studies (50/60, 83%) did not restrict their search or analysis to any named AEs, while the other 17% (10/60) of the studies named AEs (such as fever or cutaneous AEs) [44,46,56,65,68,70,84,92-94]. The extensive number of drugs and AEs included and the lack of detailed nomenclature prevented us from conducting any further analysis by drug type or AE type.

The volume of data analyzed varied between 130 to 230 million posts, whereas the volume of AEs mentions varied between 14 and 1,191,767. In general, studies that used Twitter or Facebook analyzed a larger number of posts compared with studies that used medication reviews or health forums.

**Table 2:** Characteristics of included studies (including social media platforms selected, number of drugs searched and whether named adverse events [AEs] were searched).

Category and subcategory		Studies (N=60), n (%)	References^a^
**Social media platform**			
	General social media	38 (63)	[24,35-42,44,45,48,50,52,53,55,56,58,59,61,63-68,70,73,77-81,84,85,91-95,99,104,105]
	Drug review site	9 (15)	[21,35,47,62,90,98,100-103]
	Online health forums	26 (43)	[21,24,35,36,38-43,45,46,49-52,56,57,60,69,74,76,79,82,83,85-89,94-96,98,102,103]
	Blogs	3 (5)	[56,75,85]
**Number of drugs searched**			
	1-10	33 (55)	[36-45,47,49,51,53-56,59,61-63,65-68,70-72,74-76,78,86-88,91,93,94,97,99,100,105]
	11-200	14 (23)	[21,24,35,46,48,50,57,58,64,69,73,79,92,95,102,103]
	All named	7 (12)	[60,77,89,101,104]
	Not reported	5 (8)	[52,81-83,90,96]
	Searched AEs	(1 (2)	[84]
**Only named** **AEs**			
	Yes	10 (17)	[44,46,56,65,68,70,84,92-94]
	No	(50 (83)	[21,24,35-43,45,47-55,57-64,66,67,69,71-83,85-91,95-105]

^a^Includes all publications.

### Methods of Included Studies

Seven studies [35,44,52,57,63,89,96] did not describe their methods in enough detail to identify any issues with their methodology. A further 12% (7/60) of the studies [21,24,45,50,55,56,81,95] used third-party software to detect or extract ADE mentions. For 28% (17/60) of the studies [48,51,58,64,65,69,70,75,80,82,83,85,94,97,98,102-105], some methodological issues were identified such as (1) lack of reproducibility [45], (2) no mention of manual validation of ADE mentions [58,85], (3) missing key information such as the volume of social media data from which the ADE signals were extracted or analyzed [70-72], and (4) using lexical match for ADE detection or extraction [43,48,50,58,64,69,86,89,93,98]. For the remaining 48% (29/60) studies [36-43,46,47,49,53,54,59-62,66-68,73,74,76-79,84,86-88,90-93,99-101], we did not identify any methodological issues.

Only 6 studies [36-42,45,67,82,83,93,95] mentioned that they attempted to exclude bots (or spam content) from the final set of posts, and 15 studies [21,36-42,51,53,54,61,64, 67,71,72,77,78,80,82,83,90,94,105] attempted to remove nonpersonal accounts (such as organizations or companies). Moreover, 22% (13/60) of the studies [30,36-42,53,54,58,60,61,64,68,71,72,78,79,94,105] attempted to distinguish between personal experience of the AEs from nonpersonal mentions.

### Data Source for Comparison

The most common comparison (42/60, 58%) was made with spontaneous reporting systems (such as Food and Drug Administration Adverse Event Reporting System, Medicines and Healthcare products Regulatory Agency or VigiBase). This was followed by comparisons to product labels (21/60, 29%), scientific literature (18/60, 25%), or online medical sites (5/60, 7%). Other comparisons included drug information databases, reference standards, and an internal database. Table 3 reports the details of these data sources used and their references.

**Table 3:** Data sources for adverse events compared with social media.

Data source and source name			Studies (N=60), n (%)		References
**Spontaneous reporting system**			42 (70)		—^a^
	Food and Drug Administration Adverse Event Reporting System	23 (38)		[35,45,47,56,58,61-63,67,70,76,77,79,80,90,93-95,97,99,100,102,103,105]	
	VigiBase	5 (8)		[24,50,51,60,71,72,81]	
	Medicines and Healthcare products Regulatory Agency	4 (7)		[61,65,91,92]	
	French pharmacovigilance database	3 (5)		[36-42,73,96]	
	Korea Adverse Event Reporting System	2 (3)		[75,100]	
	Vaccine Adverse Event Reporting System	2 (3)		[44,78]	
	Japanese Adverse Drug Event Report	1 (2)		[82,83]	
	MedEffect	1 (2)		[58]	
	Surveillance of Adverse Events Following Vaccination In the Community	1 (2)		[68]	
	Argentinian spontaneous reporting systems	1 (2)		[66]	
**Product labels**			21 (35)		—
	Structured Product Labeling/Summary of Product Characteristics	12 (20)		[24,36-42,45,46,49-51,53,54,56,69,73,74,98]	
	Side Effect Resource	9 (15)		[21,43,48,57,64,77,79,85,102,103]	
**Scientific literature**			18 (30)		—
	Scientific literature	7 (12)		[21,52,69,70,86-89,102,103]	
	Clinical trials	6 (10)		[53,54,59,66,67,69,86-88]	
	Systematic reviews	3 (5)		[61,67,99]	
	PubMed	2 (3)		[55,67]	
**Medical websites**			4 (7)		—
	MedlinePlus	2 (3)		[67,104]	
	Drug Bank	1 (2)		[84]	
	Drugs.com	1 (2)		[58]	
	WebMD	1 (2)		[57]	
**Other**			12 (20)		—
	Drug Information Database	4 (7)		[36-42,61,73,99]	
	Safety communications	3 (5)		[67,101]	
	Reference standards	2 (3)		[24,50,77]	
	Administrative claims	1 (2)		[56]	
	Internal adverse drug event database	1 (2)		[45]	
	Surveys	1 (2)		[53,54]	

^a^Not applicable.

### Method of Comparison

The most common method of comparing AEs was by frequency (33/60, 55%) [24,36-47,50,53,54,57,59-63,65-67,73,74,78,79, 81-83,85-92,94,96,99,105], followed by type of AEs (30/60, 50%) [16,21,30,36-42,47-49,51-54,57,58,63,64,66,70-72,77, 80-83,86-90,93,95,96,98,100,102-104], rank order of AEs (11/60, 18%) [43,45,47,53,54,61,68,75,76,78,82,83,99], and timing of AE identification (10/60, 17%) [24,35,50,71,72,79,86-88,93-95,98,101]. Other methods included disproportionality analysis, or comparing correlation and agreement, proportion, and proportional reporting ratios (15/60, 25%) [36-43,46,51,55,61,68,71,72,77,85-88,90,92, 95,99], which are used to detect more frequently reported drug-adverse drug reaction pairs or to detect potential safety signals. In addition, precision [35,92,102,103] and recall [35], among other metrics such as sensitivity, specificity, positive predictive value, and negative predictive value [56] of the detection were sometimes compared between different data sources to evaluate detection accuracy and specificity.

### Results of Comparison

Many of the publications state that similar patterns of AEs were reported in social media as compared to other traditional pharmacovigilance data sources [35-43,47,48,51-56,60-62,64-70,74-78,82,83,85-89,92,94,98,99,102-105]. However, some studies [24,45,46,50,57,59,62,89,94,102,103] detected fewer numbers of AEs on social media.

Another limitation noted of social media data was that no serious AEs were detected [36-42,45,52,61,73,77-80,82,83,91,95,96]. de Langen et al [52] noted that serious AEs were only identified in the literature.

The main advantages noted were that social media data included unexpected or new AEs [35-43,49,51,53,54,57,60,64,67,71-73, 80,84,86-90,92,98,101,104,105] (24/60, 40%) and that AEs could be identified earlier [35,60,71,72,79,86-88,92,93,98,101] (9/60, 15%) in social media as compared to those reported in spontaneous reporting systems [35,71,72,76,79,93], search query logs from search engines [35], drug safety communications [101], and scientific literature [76,86-88]. In contrast, 3 (5%) out of the 60 studies suggested that routine surveillance of social media would not aid in earlier identification of ADE signals [24,50,95], while one stated it will not be useful to confirm previously identified safety signals [45] and another one stated that certain social media platforms (such as online health forums) may be timelier in signal detection while others (Twitter) will not [35].

Regarding evaluation metrics, findings from these publications were inconsistent. One study concluded that social media had a generally higher recall but lower precision in ADE detection than other data sources such as search query logs [35]. However, this conclusion was noted to be context specific, because different social media channels had performed better or worse depending on for which event-type they were tasked to detect the signals [35]. Meanwhile, social media was also found to be more sensitive in detecting ADE than administrative claims, but less sensitive than the spontaneous reporting system of Food and Drug Administration Adverse Event Reporting System [56]. In addition, social media detection was found to be more specific, able to yield higher positive predictive value and similarly low negative predictive value as other data sources [56].

### Data and Code Availability

Only 25% (15/60) of the studies stated that their data was available: 5/15 (33%) studies [53,54,62,75,92,102,103] stated that the data would be available upon request, and the other 10/15 (67%) [24,46,49,50,58,59,61,64,65,75,77,94] studies either provided data as supplemental material or a link to a repository. In 2 cases [39,64], the links were no longer working when checked as part of this review.

Five studies [53,54,64,65,86-88] stated that their code was available. All links were validated, and one link [64] was found to no longer work.

### Author’s Conclusions

Overall, out of the selected 60 studies, 47 (78%) were supportive of the use of social media as an adjunct to traditional pharmacovigilance (Table 4). Of the rest, 8 (13%) studies stated that there may be potential value in the use of social media in pharmacovigilance, but more research is required to improve methods. Only 5 (8%) out of the 60 studies were not supportive of the use of data from social media for pharmacovigilance; however, 1 (20%) of the 5 noted that usefulness may be improved with advances in techniques used to identify ADEs in social media posts.

**Table 4:** Author’s conclusions on the use of social media for pharmacovigilance.

Author’s conclusion	Studies (N=60), n (%)	References
Support—as complementary resources	47 (78)	[21,35,44,46-49,52-61,63-68,71,72,74-76,78,81-84,86-92,96-105]
Support—with more research to improve methods	8 (13)	[36-43,51,62,73,79,80,93]
Unsupportive	4 (7)	[45,77,94,95]
Unsupportive—may be improved with more research	1 (2)	[24,50]

## Discussion

### Principal Findings

This review identified 60 studies published on the potential utility of social media in pharmacovigilance by comparing social media data to other sources since 2017. This demonstrates that the subject of using social media in AEs detection is still prolific. Indeed, many more studies were identified that analyzed social media for the purpose of identifying AEs but were done without comparison and were thus excluded from this study.

The WEB-RADR study [24,50], which is probably the most cited research on the utility of social media in pharmacovigilance, recommends that social media data not be used for broad statistical signal detection at the expense of other pharmacovigilance activities. However, the authors acknowledged several limitations with their approach, including shortcomings in their AE recognition algorithm. It was noted that the method for automatic extraction of AE mentions used in their study (primarily based on string matching) is an extremely basic approach, even for the time when the study was conducted, a choice that severely impacts the validity of their conclusion. Nonetheless, the study also noted that for certain underrepresented areas of pharmacovigilance, such as drug exposure during pregnancy, social media data could provide a valuable resource of information.

Vigi4Med project is another well-known study of social media analysis for pharmacovigilance [36-42]. This study searched for all AEs related to 6 drugs in 22 French medical forums. They extracted 60 million posts and validated 5149 posts manually. The main comparison was to the French pharmacovigilance database, although for one drug they also carried out a comparison with Summary of Product Characteristics or product labels. They concluded that although the information in forums was less informative, less serious, and contained fewer signals, it could be complementary as forums contained more unexpected AEs than the French pharmacovigilance database.

While the above 2 studies are probably the most well-known, there are a large number of other studies that analyzed the utility of social media in pharmacovigilance, as we have demonstrated.

As exemplified by these studies, the identification of ADEs and the choice of drug or comparator source can significantly influence the conclusions drawn from a study. It is crucial to consider these factors when evaluating the results. Particularly, the methods used for detecting ADEs may result in overestimation or underestimation of the reports from social media. Our findings indicate that only a few studies distinguished personal reports of ADEs from other general mentions, potentially introducing biases. While this may be less problematic in moderated patient health forums, it becomes more challenging when general social media platforms are used, where various factors can lead individuals to mention drug-related AEs that are not based on personal experiences. In addition, it is important to implement filters or rules in ADE detection to ensure that mentions are not negations, feared ADEs, or unrelated signs and symptoms, such as indications for a drug that do not represent an ADE. Failure to incorporate these measures may result in an inflated number of captured ADEs.

Detection of ADEs can be limited by certain methods. Many studies [24,43,48,50,58,64,69,71,72,89,93,98] (notably, WEB-RADR) relied on dictionary-based or lexical matching systems to identify ADE mentions. These methods may overlook a great number of mentions due to the descriptive idiomatic and nontechnical language used by patients to describe their symptoms. The lexicons used by these systems were typically curated from traditional sources such as drug labels or Side Effect Resource database (SIDER), which do not capture the full range of patient expressions. While incorporating consumer-generated terms, such as those from consumer health vocabularies or previous social media mentions, expands the number of matches, a lexical match method still primarily identifies frequently reported ADEs. In contrast, studies using advanced NLP and machine learning techniques, such as deep learning, have demonstrated superior performance in ADE recognition, including rare and previously unknown ADEs. For instance, Xia [101] developed a historical awareness multilevel framework that leverages transfer learning from prior review embeddings and uses Bidirectional Encoder Representations from Transformers–based sentence and word embeddings with an attention mechanism. This approach achieved state-of-the-art performance with an impressive *F*_1_-score of 0.944.

In several studies, it was observed that the frequency of drug mentions in social media varied depending on the specific drug [24,50,101,105]. It was reported that drugs ranked in the top 100 by sales generated more posts compared to other drugs. Therefore, the selection of drugs for study can impact the conclusions regarding the use of social media for pharmacovigilance. In addition, the use of a single comparator can introduce further issues. For instance, SIDER, a database of ADEs extracted from product labels lacks coverage for many drugs and has not been updated since 2015, potentially missing newly reported ADEs on updated labels or reported in the literature. Interestingly, 2 studies [21,43] noted that the number of new ADEs identified in social media was higher than with SIDER. However, fewer new ADEs are identified in social media if a comparison is made to more up-to-date sources such as ClinicalTrials.gov, Food and Drug Administration data, and PubMed or MEDLINEPlus [46].

### Future Research Directions

The question as to the utility of social media analysis in identifying AEs does not appear to be resolved. Future research, particularly with the advancement of artificial intelligence, should be welcomed. It may be, however, that we should not be asking social media to replace spontaneous reporting systems but more as an adjunct and to develop social media listening skills akin to those used in businesses. For example, social media is increasingly being recognized as a source for patient perspectives, and this was evident in our included studies as many studies [36-42,45-47,51-54,57,60,61,68,78,91,95,98,99] discussed the application of social media data for identifying quality of life issues, adherence behavior, or coping mechanisms [106]. Research into the value of social media to identify trends in the public discourse, public concerns, and patient perspectives could prove useful.

### Summary of and Comparison With Previous Systematic and Scoping Reviews

In our previous systematic review in 2015, we identified 29 studies comparing social media AEs data to another source of data [61]. These studies focused on using discussion forums, whereas in our review the dominant platform used was Twitter, followed by discussion forums. We now include other platforms such as Reddit and WebMD, which were not identified in our previous review. The sources used to compare against were similar to those noted in this review. Previously, we found that social media data had general agreement with other data sources for patterns of AEs but showed the potential to identify AEs earlier (one included study) and to identify new or unexpected AEs—particularly symptomatic “mild” symptoms. This agrees with this review, with more studies now investigating the timelines of social media data.

Our 2015 review [26] identified 22 technical papers on the extraction of AEs data, but such papers were excluded in our current review if they did not compare the results to an existing data source. The large number of technical papers that we excluded indicates that many more papers have been published since 2015 for the purpose of extraction. Interestingly, only 6 of 22 studies in the review by Sarker et al [26] made their annotations publicly available, a ratio comparable to our review.

The review by Lardon et al [30] focused on summarizing methods used for identifying, extracting, and evaluating the quality of medical information from social media. They found that works about identification tend to not accurately assess the completeness, quality, and reliability of the social media data being analyzed, whereas works about extraction had limited generalizability to new sites and data sources [30]. Given the limited information found through 24 publications, they concluded that the studies they reviewed were inadequate for precisely determining the role of social media data in pharmacovigilance.

Tricco et al [12] reviewed 19 studies that compared AEs reported through social media to validated data. According to Tricco et al [12], previous research showed that social media data has the potential to supplement regulatory data as they allow for earlier detection of AEs and detection of less frequently reported AEs. But Tricco et al [12] questioned the validity and reliability of these systems that use social media data for ADE detection, as none of the works they reviewed reported on these 2 important dimensions. On the basis of these findings, Tricco et al [12] concluded that the use of social media data for pharmacovigilance was “in its infancy” at the time of their reporting.

On the basis of the 38 studies reviewed by Convertino et al [27], it was found that social media data occasionally—but not always—allowed for identification of serious and unexpected proto-ADEs, but that social media was lower in information quality compared with spontaneous reporting databases, with causal relationships rarely evaluated in the detected events. Overall, Convertino et al [27] did not recommend the use of social media signal detection for routine pharmacovigilance as of the end of 2017.

Pappa and Stergioulas [28], in a more recent review of 100 articles, compared different approaches to using social media data in pharmacovigilance. They concluded that in its use for pharmacovigilance, social media data had both advantages and limitations in population coverage, usefulness, accessibility, and processability; advantages in timeliness; and limitations in quality [28]. Similar to what we found in this review, Pappa and Stergioulas [28] argued that within the big umbrella term of social media data (or social data), different types of social media data sources can vary in specific evaluative dimensions. For example, data from generic social networking sites (such as Twitter) tend to raise more quality concerns and require more quality control as compared with data from specialized health care social networks and forums (such as WebMD or What to Expect). The latter have more relevant data and lengthier postings that have the potential for broader analysis.

Lee et al [29] had a more specific focus, looking at the use of social media data in detecting new black box warnings, labeling changes, or withdrawals in advance. There were 2 studies [24,93] included in the review by Lee et al [29] that were published from 2017 onward and both these reviews are included in our scoping review. These studies were 2 of the 4 studies that reported negative or modest results. A further 9 studies in the review by Lee et al [29] were positive. This can be compared with the 10 studies in our review that measured timeliness of AEs detection, of which 9 reported positive findings.

### Limitations

The main limitations of our study are the exclusion of studies published in languages other than English, French, or Spanish and the use of Anglo-dominated databases. However, we only identified one paper in a non-English language that we could not translate and is likely to have met our inclusion criteria. This is also a fast-paced area of research, which means that the applicability of our findings may change over time. Indeed, the social media platforms themselves are rapidly changing in terms of use and access, and the technological developments to extract data from social media are rapidly evolving. The period in which each included study was undertaken, may have an impact on their findings.

It was also impossible to identify any patterns of results in relation to the type of medication studied or the types of AEs sought. This was due to a combination of poor reporting of the drug names and AEs and the large number of drugs (up to 4888) included in some studies.

As this is a scoping review, we also did not conduct any formal risk of bias assessment to ensure the validity of the results. It should be noted that any risk of bias assessment will be challenging given the lack of a validated tool for the types of studies included.

The interpretation of the results and the authors’ conclusions extracted from the included studies are subjective, the primary authors may be biased as to their initial objective, their funding, and the impact of the results on their career progression.

While we limited our review to studies with a comparison to gain a better understanding of the potential utility of social media analysis, it is important to note that utility is an ambiguous concept—what may be useful to regulatory agencies may differ to patients or clinicians for example. We should also be mindful of false positives within any system measuring case reports of AEs given that causality cannot be proven. False positives may, however, still be important to identify given the potential impact on uptake and adherence of medication.

### Conclusions

The results of this study may help inform current recommended practices and the future direction of research in this area. Most studies concluded that social media can be a useful adjunct to traditional sources. It was apparent from our study that social media data may prove most fruitful for more timely hypothesis generation of new or unexpected AEs and for detecting reports of mild symptomatic events. Knowledge of mild symptomatic events is difficult to quantify and has been shown through social media to play a role in adherence patterns [107,108] and coping strategies [106]. Future research that uses state-of-the-art NLP methods to identify personal experiences of AEs from a range of platforms and that can directly capture reports of medication change alongside the reasons for change poses to bring the best return-on-investment for the incorporation of social media data with other traditional data sources.

## Appendix

Multimedia Appendix 1 Supplementary materials.

Multimedia Appendix 2 PRISMA-ScR (Preferred Reporting Items for Systematic Reviews and Meta-Analyses Extension for Scoping Reviews) checklist.

## Abbreviations

ADE: adverse drug event
AE: adverse event
NLP: natural language processing
PRISMA-ScR: Preferred Reporting Items for Systematic Reviews and Meta-Analyses Extension for Scoping Reviews
SIDER: Side Effect Resource database

## Data Availability

All data generated or analyzed during this study are included in this published article (and its supplementary information files).
