# Supplementary material for: The Value of Social Media Analysis for Adverse Events Detection and Pharmacovigilance: Scoping Review
Source: JMIR Public Health Surveill. 2024 Sep 6;10:e59167. doi: 10.2196/59167 (PMC11415724; doi:10.2196/59167)
Supplement: Multimedia Appendix 1 [file publichealth_v10i1e59167_app1.docx]

**Supplementary Materials**

**Table S1: Description of comparator resources used in included studies.**

| **Name** | **Abbreviation** | **Description** | **Names of Resources used in Studies** |
| --- | --- | --- | --- |
| Spontaneous Reporting Systems | SRS | Systems which receive unsolicited reports of safety information about a drug, such as adverse events, from patients, providers, manufacturers, or others. These are usually maintained by government regulatory agencies. The reports are monitored to detect new signals of adverse events for a medication. | FAERS (aka FDAble, MedWatch), MHRA (aka Yellow Card), FPVD, JAERS, KAERS, Safevic, Vigibase, AAERS [KO1], VAERS |
| Drug Information Databases | DID | Compendia of referential drug information, including known safety information, used primarily as a resource by medical professionals (e.g., pharmacist, physicians). | DrugDex, Facts and Comparisons, Clinical Pharmacology, Martindale, Micromedex, Lexicomp |
| Side Effect Resource | SIDER | A database of side effects and their frequencies as reported in drug product labels. The side effects and their frequencies were automatically extracted from Structured Product Labels using Natural Language Processing Methods. | SIDER |
| Structured Product Label/Summary of Product Characteristics | SPL/SPC | Information on drug packaging or leaflet inserts that contain pertinent information about the drug. Regulatory agencies mandate the information that must be provided to consumers, such as the name of drug, indications for use, allergic reactions and side effects, and boxed warnings. | FDA DailyMed |
| Regulatory Agency Communications |  | Periodic regulatory updates on drug safety discoveries and warning that are issued prior to updating official information such as the SPL/SPC. | FDA MedWatch, FDA Drug Safety Communications |
| Clinical Trial |  | Adverse event information that was reported during a clinical trial for a medication for a certain indication | Clinicaltrials.gov |

**Table S2: Database search results**

| Resource | Platform/Interface | Date Range | Date Searched | Number of Records Retrieved |
| --- | --- | --- | --- | --- |
| MEDLINE | OVID | 1946 to February 13, 2023 | 14/02/2023 | 2132 |
| EMBASE | OVID | 1974 to 2023 February 13 | 14/02/2023 | 3708 |
| PsycINFO | OVID | 2002 to February Week 1 2023 | 14/02/2023 | 378 |
| Science Citation Index Expanded (SCI-EXPANDED) | Web of Science | 1900-present | 14/02/2023 | 1398 |
| Conference Proceedings Citation Index – Science (CPCI-S) | Web of Science | 1900-present | 14/02/2023 | 309 |
| Emerging Sources Citation Index (ESCI) | Web of Science | 1900-present | 14/02/2023 | 367 |
| Library, Information Science & Technology Abstracts (LISTA) | EBSCO | All years | 14/02/2023 | 126 |
| OpenDissertations | EBSCO | All years | 15/02/2023 | 77 |
| Proquest Dissertations & Theses: UK & Ireland | Proquest | All years | 15/02/2023 | 34 |
| ACM Digital Library | Association for Computing Machinery | All years | 15/02/2023 | 65 |
| IEEE Xplore | IEEE.org | All years | 16/02/2023 | 87 |
| Google Scholar | Internet | 2017-2023 | 17/02/2023 | 300* |
| TOTAL (before duplicates removed) |  |  |  | 8981 |
| TOTAL (after duplicates removed) |  |  |  | 5461 |
| Note: *First 300 records were sifted of 2510000 results | | | | |

**Table S3: Citation Searching using CitationChaser**

| Source |  | Total References | Unique References | Unique References Published 2017-2023 | Unique References Not Identified by Database Searching or Citation Searching |
| --- | --- | --- | --- | --- | --- |
| Related Systematic Reviews (n=29)* | Backward citation searching | 1669 | 1361 | 417 | 373 |
| Included Studies (n=63) | Backward citation searching | 1807 | 1215 | 396 | 283 |
| Included Studies (n=63) | Forward citation searching | 725 | 525 | 525 | 421 |
| TOTAL |  | 4201 | 3101 | 1338 | 1077 |
| *Excludes one thesis, one book chapter and three conference abstracts | | | | | |
| **Excludes 10 conference abstracts/dissertations not identifiable through CitationChaser | | | | | |

## **Database Search Strategies**

**Ovid MEDLINE(R) ALL <1946 to February 13, 2023>**

Searched: 14/02/2023

Records retrieved: 2132

1 social media.ti,ot,ab,kw,kf. 26781

2 social medias.ti,ot,ab,kw,kf. 136

3 web 2*.ti,ot,ab,kw,kf. 1628

4 blog*.ti,ot,ab,kw,kf. 2428

5 wiki.ti,ot,ab,kw,kf. 696

6 wikis.ti,ot,ab,kw,kf. 193

7 weblog*.ti,ot,ab,kw,kf. 144

8 web log*.ti,ot,ab,kw,kf. 73

9 facebook.ti,ot,ab,kw,kf. 6162

10 face book.ti,ot,ab,kw,kf. 12

11 tweet.ti,ot,ab,kw,kf. 903

12 tweets.ti,ot,ab,kw,kf. 2815

13 tweeting.ti,ot,ab,kw,kf. 284

14 twitter*.ti,ot,ab,kw,kf. 6229

15 you tube.ti,ot,ab,kw,kf. 22

16 youtube.ti,ot,ab,kw,kf. 3488

17 social web.ti,ot,ab,kw,kf. 73

18 social software.ti,ot,ab,kw,kf. 42

19 social medium.ti,ot,ab,kw,kf. 28

20 crowd sourcing.ti,ot,ab,kw,kf. 264

21 crowdsourcing.ti,ot,ab,kw,kf. 1760

22 instant messaging.ti,ot,ab,kw,kf. 462

23 microblogging.ti,ot,ab,kw,kf. 200

24 social bookmark*.ti,ot,ab,kw,kf. 37

25 patientslikeme.ti,ot,ab,kw,kf. 70

26 patient forum*.ti,ot,ab,kw,kf. 74

27 discussion forum*.ti,ot,ab,kw,kf. 990

28 online forum*.ti,ot,ab,kw,kf. 853

29 chat forum*.ti,ot,ab,kw,kf. 22

30 chatforum*.ti,ot,ab,kw,kf. 2

31 chatroom*.ti,ot,ab,kw,kf. 81

32 chat room*.ti,ot,ab,kw,kf. 316

33 (online adj2 discussion*).ti,ot,ab,kw,kf. 1250

34 discussion board*.ti,ot,ab,kw,kf. 433

35 online chat*.ti,ot,ab,kw,kf. 189

36 (online adj2 communication*).ti,ot,ab,kw,kf. 919

37 digital media.ti,ot,ab,kw,kf. 1226

38 digital medias.ti,ot,ab,kw,kf. 10

39 personal health message*.ti,ot,ab,kw,kf. 3

40 user comment*.ti,ot,ab,kw,kf. 125

41 patients posts.ti,ot,ab,kw,kf. 11

42 users posts.ti,ot,ab,kw,kf. 29

43 internet accounts.ti,ot,ab,kw,kf. 4

44 internet sites.ti,ot,ab,kw,kf. 568

45 message board*.ti,ot,ab,kw,kf. 209

46 web scale.ti,ot,ab,kw,kf. 40

47 google plus.ti,ot,ab,kw,kf. 10

48 user generated.ti,ot,ab,kw,kf. 682

49 consumer generated.ti,ot,ab,kw,kf. 58

50 online health content.ti,ot,ab,kw,kf. 14

51 internet narrative*.ti,ot,ab,kw,kf. 7

52 social network* site*.ti,ot,ab,kw,kf. 1713

53 online social network*.ti,ot,ab,kw,kf. 904

54 social networking.ti,ot,ab,kw,kf. 3635

55 online comment*.ti,ot,ab,kw,kf. 141

56 internet forum*.ti,ot,ab,kw,kf. 228

57 web forum*.ti,ot,ab,kw,kf. 85

58 internet media.ti,ot,ab,kw,kf. 79

59 web media.ti,ot,ab,kw,kf. 16

60 sentiment analysis.ti,ot,ab,kw,kf. 1340

61 Social Media/ 14997

62 Blogging/ 1085

63 Crowdsourcing/ 1188

64 health forum*.ti,ot,ab,kw,kf. 353

65 instagram.ti,ot,ab,kw,kf. 1651

66 linkedin.ti,ot,ab,kw,kf. 307

67 reddit.ti,ot,ab,kw,kf. 513

68 mastodon.ti,ot,ab,kw,kf. 71

69 tiktok.ti,ot,ab,kw,kf. 280

70 snapchat.ti,ot,ab,kw,kf. 202

71 spotify.ti,ot,ab,kw,kf. 24

72 pinterest.ti,ot,ab,kw,kf. 98

73 telegram.ti,ot,ab,kw,kf. 179

74 tumblr.ti,ot,ab,kw,kf. 67

75 wechat.ti,ot,ab,kw,kf. 1007

76 whatsapp.ti,ot,ab,kw,kf. 1506

77 webmd.ti,ot,ab,kw,kf. 120

78 dailystrength.ti,ot,ab,kw,kf. 6

79 askapatient.ti,ot,ab,kw,kf. 2

80 or/1-79 53918

81 (adverse adj2 (interaction$ or response$ or effect$ or event$ or reaction$ or outcome$)).ti,ot,ab,kw,kf. 566367

82 side effect$.ti,ot,ab,kw,kf. 297650

83 (unintended adj2 (interaction$ or response$ or effect$ or event$ or reaction$ or outcome$)).ti,ot,ab,kw,kf. 2168

84 (unintentional adj2 (interaction$ or response$ or effect$ or event$ or reaction$ or outcome$)).ti,ot,ab,kw,kf. 315

85 (unwanted adj2 (interaction$ or response$ or effect$ or event$ or reaction$ or outcome$)).ti,ot,ab,kw,kf. 7742

86 (unexpected adj2 (interaction$ or response$ or effect$ or event$ or reaction$ or outcome$)).ti,ot,ab,kw,kf. 8108

87 (undesirable adj2 (interaction$ or response$ or effect$ or event$ or reaction$ or outcome$)).ti,ot,ab,kw,kf. 10035

88 (serious adj2 (interaction$ or response$ or effect$ or event$ or reaction$ or outcome$)).ti,ot,ab,kw,kf. 52585

89 (toxic adj2 (interaction$ or response$ or effect$ or event$ or reaction$ or outcome$)).ti,ot,ab,kw,kf. 65877

90 (adrs or ades).ti,ot,ab,kw,kf. 6907

91 drug safety.ti,ot,ab,kw,kf. 6591

92 (drug surveillance or ((postmarketing or post marketing) adj2 surveillance)).ti,ot,ab,kw,kf. 3922

93 product surveillance.ti,ot,ab,kw,kf. 144

94 drug monitoring.ti,ot,ab,kw,kf. 13514

95 tolerability.ti,ot,ab,kw,kf. 57842

96 treatment emergent.ti,ot,ab,kw,kf. 6999

97 toxicity.ti,ot,ab,kw,kf. 461454

98 pharmacovigilance.ti,ot,ab,kw,kf. 7264

99 drug withdrawal*.ti,ot,ab,kw,kf. 4342

100 ae.fs. 1965509

101 to.fs. 475711

102 Product Surveillance, Postmarketing/ 7594

103 Adverse Drug Reaction Reporting Systems/ 8717

104 pharmacovigilance/ 3181

105 Drug Monitoring/ 23409

106 exp Drug Hypersensitivity/ 49341

107 exp "Drug-Related Side Effects and Adverse Reactions"/ 130275

108 Abnormalities, Drug-Induced/ 14733

109 Safety-Based Drug Withdrawals/ 415

110 Drug Recalls/ 156

111 safety signal*.ti,ot,ab,kw,kf. 3037

112 or/81-111 3292946

113 80 and 112 2804

114 limit 113 to yr="2017 -Current" 2132

**Embase <1974 to 2023 February 13>**

Searched: 14/02/2023

Records retrieved: 3708

1 social media.ti,ot,ab,kw,kf. 33700

2 social medias.ti,ot,ab,kw,kf. 185

3 web 2*.ti,ot,ab,kw,kf. 1897

4 blog*.ti,ot,ab,kw,kf. 3779

5 wiki.ti,ot,ab,kw,kf. 936

6 wikis.ti,ot,ab,kw,kf. 251

7 weblog*.ti,ot,ab,kw,kf. 182

8 web log*.ti,ot,ab,kw,kf. 98

9 facebook.ti,ot,ab,kw,kf. 8942

10 face book.ti,ot,ab,kw,kf. 73

11 tweet.ti,ot,ab,kw,kf. 1129

12 tweets.ti,ot,ab,kw,kf. 3012

13 tweeting.ti,ot,ab,kw,kf. 377

14 twitter*.ti,ot,ab,kw,kf. 7800

15 you tube.ti,ot,ab,kw,kf. 146

16 youtube.ti,ot,ab,kw,kf. 4507

17 social web.ti,ot,ab,kw,kf. 89

18 social software.ti,ot,ab,kw,kf. 57

19 social medium.ti,ot,ab,kw,kf. 35

20 crowd sourcing.ti,ot,ab,kw,kf. 352

21 crowdsourcing.ti,ot,ab,kw,kf. 1899

22 instant messaging.ti,ot,ab,kw,kf. 591

23 microblogging.ti,ot,ab,kw,kf. 225

24 social bookmark*.ti,ot,ab,kw,kf. 47

25 patientslikeme.ti,ot,ab,kw,kf. 151

26 patient forum*.ti,ot,ab,kw,kf. 177

27 discussion forum*.ti,ot,ab,kw,kf. 1429

28 online forum*.ti,ot,ab,kw,kf. 1217

29 chat forum*.ti,ot,ab,kw,kf. 46

30 chatforum*.ti,ot,ab,kw,kf. 3

31 chatroom*.ti,ot,ab,kw,kf. 117

32 chat room*.ti,ot,ab,kw,kf. 434

33 (online adj2 discussion*).ti,ot,ab,kw,kf. 1668

34 discussion board*.ti,ot,ab,kw,kf. 638

35 online chat*.ti,ot,ab,kw,kf. 272

36 (online adj2 communication*).ti,ot,ab,kw,kf. 1093

37 digital media.ti,ot,ab,kw,kf. 1476

38 digital medias.ti,ot,ab,kw,kf. 9

39 personal health message*.ti,ot,ab,kw,kf. 3

40 user comment*.ti,ot,ab,kw,kf. 141

41 patients posts.ti,ot,ab,kw,kf. 17

42 users posts.ti,ot,ab,kw,kf. 24

43 internet accounts.ti,ot,ab,kw,kf. 7

44 internet sites.ti,ot,ab,kw,kf. 773

45 message board*.ti,ot,ab,kw,kf. 314

46 web scale.ti,ot,ab,kw,kf. 38

47 google plus.ti,ot,ab,kw,kf. 25

48 user generated.ti,ot,ab,kw,kf. 691

49 consumer generated.ti,ot,ab,kw,kf. 62

50 online health content.ti,ot,ab,kw,kf. 14

51 internet narrative*.ti,ot,ab,kw,kf. 16

52 social network* site*.ti,ot,ab,kw,kf. 2049

53 online social network*.ti,ot,ab,kw,kf. 977

54 social networking.ti,ot,ab,kw,kf. 4546

55 online comment*.ti,ot,ab,kw,kf. 164

56 internet forum*.ti,ot,ab,kw,kf. 364

57 web forum*.ti,ot,ab,kw,kf. 113

58 internet media.ti,ot,ab,kw,kf. 109

59 web media.ti,ot,ab,kw,kf. 20

60 sentiment analysis.ti,ot,ab,kw,kf. 1218

61 Social Media/ 43172

62 Blogging/ 731

63 Crowdsourcing/ 2195

64 health forum*.ti,ot,ab,kw,kf. 398

65 instagram.ti,ot,ab,kw,kf. 2253

66 linkedin.ti,ot,ab,kw,kf. 561

67 reddit.ti,ot,ab,kw,kf. 599

68 mastodon.ti,ot,ab,kw,kf. 44

69 tiktok.ti,ot,ab,kw,kf. 338

70 snapchat.ti,ot,ab,kw,kf. 261

71 spotify.ti,ot,ab,kw,kf. 49

72 pinterest.ti,ot,ab,kw,kf. 144

73 telegram.ti,ot,ab,kw,kf. 232

74 tumblr.ti,ot,ab,kw,kf. 82

75 wechat.ti,ot,ab,kw,kf. 1079

76 whatsapp.ti,ot,ab,kw,kf. 2236

77 webmd.ti,ot,ab,kw,kf. 295

78 dailystrength.ti,ot,ab,kw,kf. 6

79 askapatient.ti,ot,ab,kw,kf. 0

80 or/1-79 76332

81 (adverse adj2 (interaction$ or response$ or effect$ or event$ or reaction$ or outcome$)).ti,ot,ab,kw,kf. 897481

82 side effect$.ti,ot,ab,kw,kf. 436497

83 (unintended adj2 (interaction$ or response$ or effect$ or event$ or reaction$ or outcome$)).ti,ot,ab,kw,kf. 2629

84 (unintentional adj2 (interaction$ or response$ or effect$ or event$ or reaction$ or outcome$)).ti,ot,ab,kw,kf. 414

85 (unwanted adj2 (interaction$ or response$ or effect$ or event$ or reaction$ or outcome$)).ti,ot,ab,kw,kf. 10333

86 (unexpected adj2 (interaction$ or response$ or effect$ or event$ or reaction$ or outcome$)).ti,ot,ab,kw,kf. 11107

87 (undesirable adj2 (interaction$ or response$ or effect$ or event$ or reaction$ or outcome$)).ti,ot,ab,kw,kf. 13350

88 (serious adj2 (interaction$ or response$ or effect$ or event$ or reaction$ or outcome$)).ti,ot,ab,kw,kf. 85251

89 (toxic adj2 (interaction$ or response$ or effect$ or event$ or reaction$ or outcome$)).ti,ot,ab,kw,kf. 82009

90 (adrs or ades).ti,ot,ab,kw,kf. 13144

91 drug safety.ti,ot,ab,kw,kf. 11244

92 (drug surveillance or ((postmarketing or post marketing) adj2 surveillance)).ti,ot,ab,kw,kf. 6272

93 product surveillance.ti,ot,ab,kw,kf. 335

94 drug monitoring.ti,ot,ab,kw,kf. 21892

95 tolerability.ti,ot,ab,kw,kf. 108508

96 treatment emergent.ti,ot,ab,kw,kf. 17752

97 toxicity.ti,ot,ab,kw,kf. 642989

98 pharmacovigilance.ti,ot,ab,kw,kf. 15014

99 drug withdrawal*.ti,ot,ab,kw,kf. 6449

100 ae.fs. 1325517

101 to.fs. 581822

102 safety signal*.ti,ot,ab,kw,kf. 8294

103 si.fs. 974439

104 exp adverse drug reaction/ 622853

105 exp drug toxicity/ 157054

106 drug monitoring/ 58666

107 exp postmarketing surveillance/ 38715

108 exp drug hypersensitivity/ 76533

109 drug recall/ 757

110 exp drug safety/ 539921

111 exp side effect/ 669199

112 or/81-111 3771703

113 80 and 112 4931

114 limit 113 to yr="2017 -Current" 3708

**APA PsycInfo <2002 to February Week 1 2023>**

Searched: 14/02/2023

Records retrieved: 378

1 social media.ti,ot,ab,hw,id. 22152

2 social medias.ti,ot,ab,hw,id. 176

3 web 2*.ti,ot,ab,hw,id. 1519

4 blog*.ti,ot,ab,hw,id. 3818

5 wiki.ti,ot,ab,hw,id. 478

6 wikis.ti,ot,ab,hw,id. 468

7 weblog*.ti,ot,ab,hw,id. 249

8 web log*.ti,ot,ab,hw,id. 89

9 facebook.ti,ot,ab,hw,id. 7060

10 face book.ti,ot,ab,hw,id. 20

11 tweet.ti,ot,ab,hw,id. 428

12 tweets.ti,ot,ab,hw,id. 1566

13 tweeting.ti,ot,ab,hw,id. 222

14 twitter*.ti,ot,ab,hw,id. 3969

15 you tube.ti,ot,ab,hw,id. 30

16 youtube.ti,ot,ab,hw,id. 1619

17 social web.ti,ot,ab,hw,id. 153

18 social software.ti,ot,ab,hw,id. 140

19 social medium.ti,ot,ab,hw,id. 51

20 crowd sourcing.ti,ot,ab,hw,id. 133

21 crowdsourcing.ti,ot,ab,hw,id. 1276

22 instant messaging.ti,ot,ab,hw,id. 746

23 microblogging.ti,ot,ab,hw,id. 256

24 social bookmark*.ti,ot,ab,hw,id. 55

25 patientslikeme.ti,ot,ab,hw,id. 23

26 patient forum*.ti,ot,ab,hw,id. 11

27 discussion forum*.ti,ot,ab,hw,id. 1113

28 online forum*.ti,ot,ab,hw,id. 921

29 chat forum*.ti,ot,ab,hw,id. 22

30 chatforum*.ti,ot,ab,hw,id. 0

31 chatroom*.ti,ot,ab,hw,id. 125

32 chat room*.ti,ot,ab,hw,id. 570

33 (online adj2 discussion*).ti,ot,ab,hw,id. 2024

34 discussion board*.ti,ot,ab,hw,id. 659

35 online chat*.ti,ot,ab,hw,id. 342

36 (online adj2 communication*).ti,ot,ab,hw,id. 1730

37 digital media.ti,ot,ab,hw,id. 2632

38 digital medias.ti,ot,ab,hw,id. 21

39 personal health message*.ti,ot,ab,hw,id. 1

40 user comment*.ti,ot,ab,hw,id. 144

41 patients posts.ti,ot,ab,hw,id. 2

42 users posts.ti,ot,ab,hw,id. 15

43 internet accounts.ti,ot,ab,hw,id. 3

44 internet sites.ti,ot,ab,hw,id. 332

45 message board*.ti,ot,ab,hw,id. 325

46 web scale.ti,ot,ab,hw,id. 19

47 google plus.ti,ot,ab,hw,id. 14

48 user generated.ti,ot,ab,hw,id. 1136

49 consumer generated.ti,ot,ab,hw,id. 146

50 online health content.ti,ot,ab,hw,id. 7

51 internet narrative*.ti,ot,ab,hw,id. 7

52 social network* site*.ti,ot,ab,hw,id. 3928

53 online social network*.ti,ot,ab,hw,id. 9902

54 social networking.ti,ot,ab,hw,id. 5898

55 online comment*.ti,ot,ab,hw,id. 263

56 internet forum*.ti,ot,ab,hw,id. 220

57 web forum*.ti,ot,ab,hw,id. 103

58 internet media.ti,ot,ab,hw,id. 56

59 web media.ti,ot,ab,hw,id. 17

60 sentiment analysis.ti,ot,ab,hw,id. 927

61 exp Social Media/ 21255

62 Crowdsourcing/ 854

63 health forum*.ti,ot,ab,hw,id. 87

64 instagram.ti,ot,ab,hw,id. 1212

65 linkedin.ti,ot,ab,hw,id. 436

66 reddit.ti,ot,ab,hw,id. 304

67 mastodon.ti,ot,ab,hw,id. 3

68 tiktok.ti,ot,ab,hw,id. 75

69 snapchat.ti,ot,ab,hw,id. 225

70 spotify.ti,ot,ab,hw,id. 37

71 pinterest.ti,ot,ab,hw,id. 104

72 telegram.ti,ot,ab,hw,id. 42

73 tumblr.ti,ot,ab,hw,id. 85

74 wechat.ti,ot,ab,hw,id. 298

75 whatsapp.ti,ot,ab,hw,id. 391

76 webmd.ti,ot,ab,hw,id. 36

77 dailystrength.ti,ot,ab,hw,id. 0

78 askapatient.ti,ot,ab,hw,id. 0

79 or/1-78 47468

80 (adverse adj2 (interaction$ or response$ or effect$ or event$ or reaction$ or outcome$)).ti,ot,ab,hw,id. 41195

81 side effect$.ti,ot,ab,hw,id. 38539

82 (unintended adj2 (interaction$ or response$ or effect$ or event$ or reaction$ or outcome$)).ti,ot,ab,hw,id. 898

83 (unintentional adj2 (interaction$ or response$ or effect$ or event$ or reaction$ or outcome$)).ti,ot,ab,hw,id. 98

84 (unwanted adj2 (interaction$ or response$ or effect$ or event$ or reaction$ or outcome$)).ti,ot,ab,hw,id. 802

85 (unexpected adj2 (interaction$ or response$ or effect$ or event$ or reaction$ or outcome$)).ti,ot,ab,hw,id. 1937

86 (undesirable adj2 (interaction$ or response$ or effect$ or event$ or reaction$ or outcome$)).ti,ot,ab,hw,id. 1121

87 (serious adj2 (interaction$ or response$ or effect$ or event$ or reaction$ or outcome$)).ti,ot,ab,hw,id. 3647

88 (toxic adj2 (interaction$ or response$ or effect$ or event$ or reaction$ or outcome$)).ti,ot,ab,hw,id. 1368

89 (adrs or ades).ti,ot,ab,hw,id. 452

90 drug safety.ti,ot,ab,hw,id. 1339

91 (drug surveillance or ((postmarketing or post marketing) adj2 surveillance)).ti,ot,ab,hw,id. 242

92 product surveillance.ti,ot,ab,hw,id. 7

93 drug monitoring.ti,ot,ab,hw,id. 803

94 tolerability.ti,ot,ab,hw,id. 6925

95 treatment emergent.ti,ot,ab,hw,id. 1286

96 toxicity.ti,ot,ab,hw,id. 8458

97 pharmacovigilance.ti,ot,ab,hw,id. 337

98 drug withdrawal*.ti,ot,ab,hw,id. 3161

99 safety signal*.ti,ot,ab,hw,id. 342

100 "side effects (drug)"/ 17024

101 drug sensitivity/ 1199

102 tardive dyskinesia/ 798

103 neuroleptic malignant syndrome/ 481

104 "Side Effects (Treatment)"/ 2664

105 or/80-104 93294

106 79 and 105 521

107 limit 106 to yr="2017 -Current" 378

**OpenDissertations and Library, Information Science & Technology Abstracts (LISTA) on EBSCO**

Searched: 15/02/2023

Records retrieved: 77 (OpenDissertations), 126 (LISTA)

(“social media” OR “social medias” OR blog* OR wiki OR wikis OR weblog* OR “web log*” OR facebook OR “face book” OR tweet OR tweets OR tweeting OR twitter* OR “you tube” OR youtube OR “social web” OR “social software” OR “social medium” OR “crowd sourcing” OR crowdsourcing OR “instant messaging” OR microblogging OR “social bookmark*” OR patientslikeme OR “patient forum*” OR “discussion forum*” OR “online forum*” OR “chat forum*” OR chatforum OR chatroom* OR “chat room*” OR “discussion board*” OR “online chat*” OR “digital media” OR “digital medias” OR “personal health message*” OR “user comment*” OR “patients posts” OR “user posts” OR “internet accounts” OR “internet sites” OR “google plus” OR “user generated” OR “consumer generated” OR “online health content” OR “internet narrative*” OR “social network* site*” OR “social networking” OR “online comment*” OR “internet forum*” OR “web forum*” OR “internet media” OR “web media” OR “sentiment analysis” OR (online N2 discussion*) OR (online N2 communication*) OR ”web 2.0” OR “health forum*” OR instagram OR linkedin OR reddit OR mastodon OR tiktok OR snapchat OR spotify OR pinterest OR telegram OR tumblr OR wechat OR whatsapp OR webmd OR dailystrength OR askapatient) in all fields

AND

((adverse W2 interaction*) OR (adverse W2 response*) OR (adverse W2 effect*) OR (adverse W2 event*) OR (adverse W2 reaction*) OR (adverse W2 outcome*) OR (unintended W2 interaction*) OR (unintended W2 response*) OR (unintended W2 effect*) OR (unintended W2 event*) OR (unintended W2 reaction*) OR (unintended W2 outcome*) OR (unintentional W2 interaction*) OR (unintentional W2 response*) OR (unintentional W2 effect*) OR (unintentional W2 event*) OR (unintentional W2 reaction*) OR (unintentional W2 outcome*) OR (unwanted W2 interaction*) OR (unwanted W2 response*) OR (unwanted W2 effect*) OR (unwanted W2 event*) OR (unwanted W2 reaction*) OR (unwanted W2 outcome*) OR (unexpected W2 interaction*) OR (unexpected W2 response*) OR (unexpected W2 effect*) OR (unexpected W2 event*) OR (unexpected W2 reaction*) OR (unexpected W2 outcome*) OR (undesirable W2 interaction*) OR (undesirable W2 response*) OR (undesirable W2 effect*) OR (undesirable W2 event*) OR (undesirable W2 reaction*) OR (undesirable W2 outcome*) OR (serious W2 interaction*) OR (serious W2 response*) OR (serious W2 effect*) OR (serious W2 event*) OR (serious W2 reaction*) OR (serious W2 outcome*) OR (toxic W2 interaction*) OR (toxic W2 response*) OR (toxic W2 effect*) OR (toxic W2 event*) OR (toxic W2 reaction*) OR (toxic W2 outcome*) OR “side effect*” OR adrs OR ades OR “drug safety” OR “drug surveillance” OR “product surveillance” OR “drug monitoring” OR tolerability OR “treatment emergent” OR toxicity OR pharmacovigilance OR “drug withdrawal*” OR “drug recall*” OR (postmarketing N2 surveillance) OR (post N1 marketing N2 surveillance) OR “safety signal*”) in all fields

Limit to publication date 2017 onwards

**ProQuest Dissertations & Theses: UK & Ireland**

Searched: 15/02/2023

Records retrieved: 34

(“social media” OR “social medias” OR blog* OR wiki OR wikis OR weblog* OR “web log*” OR facebook OR “face book” OR tweet OR tweets OR tweeting OR twitter* OR “you tube” OR youtube OR “social web” OR “social software” OR “social medium” OR “crowd sourcing” OR crowdsourcing OR “instant messaging” OR microblogging OR “social bookmark*” OR patientslikeme OR “patient forum*” OR “discussion forum*” OR “online forum*” OR “chat forum*” OR chatforum OR chatroom* OR “chat room*” OR “discussion board*” OR “online chat*” OR “digital media” OR “digital medias” OR “personal health message*” OR “user comment*” OR “patients posts” OR “user posts” OR “internet accounts” OR “internet sites” OR “google plus” OR “user generated” OR “consumer generated” OR “online health content” OR “internet narrative*” OR “social network* site*” OR “social networking” OR “online comment*” OR “internet forum*” OR “web forum*” OR “internet media” OR “web media” OR “sentiment analysis” OR (online NEAR/2 discussion*) OR (online NEAR/2 communication*) OR ”web 2.0” OR “health forum*” OR instagram OR linkedin OR reddit OR mastodon OR tiktok OR snapchat OR spotify OR pinterest OR telegram OR tumblr OR wechat OR whatsapp OR webmd OR dailystrength OR askapatient) in title or abstract

AND

((adverse NEAR/2 interaction*) OR (adverse NEAR/2 response*) OR (adverse NEAR/2 effect*) OR (adverse NEAR/2 event*) OR (adverse NEAR/2 reaction*) OR (adverse NEAR/2 outcome*) OR (unintended NEAR/2 interaction*) OR (unintended NEAR/2 response*) OR (unintended NEAR/2 effect*) OR (unintended NEAR/2 event*) OR (unintended NEAR/2 reaction*) OR (unintended NEAR/2 outcome*) OR (unintentional NEAR/2 interaction*) OR (unintentional NEAR/2 response*) OR (unintentional NEAR/2 effect*) OR (unintentional NEAR/2 event*) OR (unintentional NEAR/2 reaction*) OR (unintentional NEAR/2 outcome*) OR (unwanted NEAR/2 interaction*) OR (unwanted NEAR/2 response*) OR (unwanted NEAR/2 effect*) OR (unwanted NEAR/2 event*) OR (unwanted NEAR/2 reaction*) OR (unwanted NEAR/2 outcome*) OR (unexpected NEAR/2 interaction*) OR (unexpected NEAR/2 response*) OR (unexpected NEAR/2 effect*) OR (unexpected NEAR/2 event*) OR (unexpected NEAR/2 reaction*) OR (unexpected NEAR/2 outcome*) OR (undesirable NEAR/2 interaction*) OR (undesirable NEAR/2 response*) OR (undesirable NEAR/2 effect*) OR (undesirable NEAR/2 event*) OR (undesirable NEAR/2 reaction*) OR (undesirable NEAR/2 outcome*) OR (serious NEAR/2 interaction*) OR (serious NEAR/2 response*) OR (serious NEAR/2 effect*) OR (serious NEAR/2 event*) OR (serious NEAR/2 reaction*) OR (serious NEAR/2 outcome*) OR (toxic NEAR/2 interaction*) OR (toxic NEAR/2 response*) OR (toxic NEAR/2 effect*) OR (toxic NEAR/2 event*) OR (toxic NEAR/2 reaction*) OR (toxic NEAR/2 outcome*) OR “side effect*” OR adrs OR ades OR “drug safety” OR “drug surveillance” OR “product surveillance” OR “drug monitoring” OR tolerability OR “treatment emergent” OR toxicity OR pharmacovigilance OR “drug withdrawal*” OR (postmarketing NEAR/2 surveillance) OR (post NEAR/1 marketing NEAR/2 surveillance) OR (safety NEAR/2 signal*)) in title

**Conference Proceedings Citation Index- Science (CPCI-S) --1990-present**

**Science Citation Index Expanded (SCI-EXPANDED)**

**Emerging Sources Citation Index (ESCI)**

Searched: 14/02/2023

Records retrieved: 309 (CPCI-S), 1398 (SCI-EXPANDED), 367 (ESCI)

#1 TS=(“social media” OR “social medias” OR blog* OR wiki OR wikis OR weblog* OR “web log*” OR facebook OR “face book” OR tweet OR tweets OR tweeting OR twitter* OR “you tube” OR youtube OR “social web” OR “social software” OR “social medium” OR “crowd sourcing” OR crowdsourcing OR “instant messaging” OR microblogging OR “social bookmark*” OR patientslikeme OR “patient forum*” OR “discussion forum*” OR “online forum*” OR “chat forum*” OR chatforum OR chatroom* OR “chat room*” OR “discussion board*” OR “online chat*” OR “digital media” OR “digital medias” OR “personal health message*” OR “user comment*” OR “patients posts” OR “user posts” OR “internet accounts” OR “internet sites” OR “google plus” OR “user generated” OR “consumer generated” OR “online health content” OR “internet narrative*” OR “social network* site*” OR “social networking” OR “online comment*” OR “internet forum*” OR “web forum*” OR “internet media” OR “web media” OR “sentiment analysis” OR (online NEAR/2 discussion*) OR (online NEAR/2 communication*) OR ”web 2.0” OR “health forum*” OR instagram OR linkedin OR reddit OR mastodon OR tiktok OR snapchat OR spotify OR pinterest OR telegram OR tumblr OR wechat OR whatsapp OR webmd OR dailystrength OR askapatient)

AND

#2 TS=((adverse NEAR/2 interaction*) OR (adverse NEAR/2 response*) OR (adverse NEAR/2 effect*) OR (adverse NEAR/2 event*) OR (adverse NEAR/2 reaction*) OR (adverse NEAR/2 outcome*) OR (unintended NEAR/2 interaction*) OR (unintended NEAR/2 response*) OR (unintended NEAR/2 effect*) OR (unintended NEAR/2 event*) OR (unintended NEAR/2 reaction*) OR (unintended NEAR/2 outcome*) OR (unintentional NEAR/2 interaction*) OR (unintentional NEAR/2 response*) OR (unintentional NEAR/2 effect*) OR (unintentional NEAR/2 event*) OR (unintentional NEAR/2 reaction*) OR (unintentional NEAR/2 outcome*) OR (unwanted NEAR/2 interaction*) OR (unwanted NEAR/2 response*) OR (unwanted NEAR/2 effect*) OR (unwanted NEAR/2 event*) OR (unwanted NEAR/2 reaction*) OR (unwanted NEAR/2 outcome*) OR (unexpected NEAR/2 interaction*) OR (unexpected NEAR/2 response*) OR (unexpected NEAR/2 effect*) OR (unexpected NEAR/2 event*) OR (unexpected NEAR/2 reaction*) OR (unexpected NEAR/2 outcome*) OR (undesirable NEAR/2 interaction*) OR (undesirable NEAR/2 response*) OR (undesirable NEAR/2 effect*) OR (undesirable NEAR/2 event*) OR (undesirable NEAR/2 reaction*) OR (undesirable NEAR/2 outcome*) OR (serious NEAR/2 interaction*) OR (serious NEAR/2 response*) OR (serious NEAR/2 effect*) OR (serious NEAR/2 event*) OR (serious NEAR/2 reaction*) OR (serious NEAR/2 outcome*) OR (toxic NEAR/2 interaction*) OR (toxic NEAR/2 response*) OR (toxic NEAR/2 effect*) OR (toxic NEAR/2 event*) OR (toxic NEAR/2 reaction*) OR (toxic NEAR/2 outcome*) OR “side effect*” OR adrs OR ades OR “drug safety” OR “drug surveillance” OR “product surveillance” OR “drug monitoring” OR tolerability OR “treatment emergent” OR toxicity OR pharmacovigilance OR “drug withdrawal*” OR (postmarketing NEAR/2 surveillance) OR (post NEAR/1 marketing NEAR/2 surveillance) OR “safety signal*”)

#2 AND #1

Publication date limit 2017 onwards

**Google Scholar**

Searched: 17/02/2023

Records retrieved: 2,510,000

Records screened: 200 (on first 20 pages)

A simplified search strategy was applied in Google Scholar due to the nature of its interface and search capabilities.

("social media" OR Twitter) AND (adverse OR "side effects" OR pharmacovigilance) Limited to 2017 to 2023

**ACM Digital Library**

Searched: 15/02/2023

Results: 65

(“social media” OR “social medias” OR blog* OR wiki OR wikis OR weblog* OR “web log” OR “web log” OR “web logs” OR facebook OR “face book” OR tweet OR tweets OR tweeting OR twitter* OR “you tube” OR youtube OR “social web” OR “social software” OR “social medium” OR “crowd sourcing” OR crowdsourcing OR “instant messaging” OR microblogging OR “social bookmark” OR “social bookmarks” OR patientslikeme OR “patient forum” OR “patient forums” OR “discussion forum” OR “discussion forums” OR “online forum” OR “online forums” OR “chat forum” OR “chat forums” OR chatforum* OR chatroom* OR “chat room” OR “chat rooms” OR “discussion board” OR “discussion boards” OR “online chat” OR “online chats” OR “digital media” OR “digital medias” OR “personal health messages” OR “user comments” OR “patients posts” OR “user posts” OR “internet accounts” OR “internet sites” OR “google plus” OR “user generated” OR “consumer generated” OR “online health content” OR “internet narrative” OR “internet narratives” OR “social network site” OR “social network sites” OR “social networking” OR “online comment” OR “online comments” OR “internet forum” OR “internet forums” OR “web forum” OR “web forums” OR “internet media” OR “web media” OR “sentiment analysis” OR “online discussion” OR “online communication” OR ”web 2.0” OR “health forum” OR “health forums” OR instagram OR linkedin OR reddit OR mastodon OR tiktok OR snapchat OR spotify OR pinterest OR telegram OR tumblr OR wechat OR whatsapp OR webmd OR dailystrength OR askapatient)

AND

(adverse OR “side effects” OR adrs OR ades OR “drug safety” OR “drug surveillance” OR “product surveillance” OR “drug monitoring” OR tolerability OR “treatment emergent” OR “drug toxicity” OR pharmacovigilance OR “drug withdrawal” OR “drug withdrawals” OR “drug recall” OR “drug recalls” OR “postmarketing surveillance” OR “post marketing surveillance” OR “safety signal” OR “safety signals”)

#2 AND #1 in title or abstract

Publication date limit 2017 onwards

**IEEE Xplore**

Searched: 16/02/2023

Results: 87

("Document Title":adverse OR "Document Title":pharmacovigilance OR "Document Title":surveillance OR "Document Title":drug OR "Document Title":safety) AND ("All Metadata":"social media" OR "Document Title":twitter OR "Document Title":facebook OR "Document Title":forum* OR "Document Title":chat OR "Document Title":reddit)

Publication date limit 2017 onwards

Search for: remove duplicates from 14 [limit 13 to yr="2016 -Current"]

**S4: Data extracted from all the included publications**

| **Publication** | **Medications Searched** | **Adverse Events Searched** | **Describe any issues** | **Removed bots?** | **Removed non-personal accounts (e.g., business, organization)?** | **Distinguish personal from non-personal mentions of AEs?** | **Volume of Data Analysed** | **Volume of AEs** | **Comparison Source Category** | **Comparison Method Category** | **Main Results (including frequency of AEs detected)** | **Authors conclusions** | **Data Available** | **Code Available** |  |
| --- | --- | --- | --- | --- | --- | --- | --- | --- | --- | --- | --- | --- | --- | --- | --- |
| Abbasi 2019[1] | 133 unique medications | All; Named - 143 drug-event pairs | Not reported in enough detail. Study gives no details on AEs detected in SM. Data were automatically tagged, no mention of validation. | No | No | No | 12 million tweets, 5 million Forum | 143 Events | SRS  Safety Communication | Timing | Analysis framework reveals that user-generated content channels can facilitate timelier detection of adverse events: on average, two to three years or earlier than commonly used databases. Additionally, we find social media channels provide higher detection rates but lower precision than do search-based signals. The search and web forum channels are timelier than Twitter. | The earlier detection rates for many events, across models and user-generated channels (i.e., often one to three years earlier), is also an interesting result that is consistent with some recent studies. Social media channels provided higher recall but lower precision than did search. Within social media, forums and Twitter each performed better on certain event types (e.g., Forums attained higher recall for product recall events in both the health and automotive test beds). Forums and search also yielded timelier detection than did Twitter | No | No |  |
| Karapetiantz 2019[2] | strontium ranelate (osteoporosis) | All | Not reported in enough detail. Details in prior publication | No | Yes | Yes | 6,569,555 posts | 55,350,564 drug/AE pairs | SRS | Frequency  Proportion | Only two serious cases were found in forums against 203 in the FPVD (4.3% vs 46.5%, p<0.0001). No death was identified in forums whereas 7 were found in the FPVD. There was no significant difference between the two sources for the number of cases containing at least one unexpected ADR (25.5% in forums vs 22.1% in the FPVD). Only three PT terms identified in forums were not reported in the FPVD: “Weight increased”, “depression” and “hypertension”. There were more SOCs describing the ADRs in the FPVD than in forums (22 vs 11) which can explain the fact that the four first SOCs in the FPVD represented only 60% of the ADRs found (487 among 840) whereas they represented 72% of the ADRs found in the forums (79 among 109). The four SOCs most represented in the FPVD were “Skin and subcutaneous tissue disorders” (31%), “Gastrointestinal disorders” (13%), “Respiratory, thoracic and mediastinal disorders” (10%), and “General disorders and administration site conditions” (8%). In forums, the four most represented SOCs were “Nervous system disorders” (22%), “Gastrointestinal disorders” (21%), “Musculoskeletal and connective tissue disorders” (16%), and the SOCs “General disorders and administration site conditions” and “Skin and subcutaneous tissue disorders” (both representing 14% of the ADRs). Thus, SOC’s distribution was significantly different between the FPVD and the forums (p < 0.0001), SOCs’ distribution being more diverse in the FPVD. | Future work will focus on implementing other statistical methods, exploring the complementarity of both approaches on a larger scale, and prioritizing the posts to manually evaluate after applying appropriate signal detection methods. | No | No |  |
| Bellet 2018* [3] | agomelatine, baclofen, duloxetine, exenatide, strontium ranelate and tetrazepam | All | Not reported in enough detail. Abstract; Manual evaluation of posts | No | Yes | Yes | 5149 | 3,001 | SRS SPL/SPC | Type | These 1,284 cases, corresponding to 3,001 ADRs (2.3 ADRs per case), were compared to 2,521 cases from the FPVD, corresponding to 5,262 ADRs (2.1 ADRs per case). Forums’ cases were significantly less informative than those of the FPVD for patients’ age and sex, dose and duration of the treatment, and time-to-onset and evolution of the ADR, but not for drug indication. They were less often serious than those registered in the FPVD (4.2% vs 45.6%). The MedDRA System Organ Classes’ distribution was different between both data sources, with a global profile of ADRs more “richer” in the FPVD. In contrast, forums reported more often unexpected ADRs (24.2% vs 17.1%, P < 0.05), including197 that were not identified in the FPVD. | This study showed a qualitative difference in the ADRs profile of drugs between forums and FPVD and confirmed the possibility to detect unexpected ADRs in patients’ posts. Despite less informative, forums could be a complementary source of knowledge for PV due to the volume of data. | No | No |  |
| Karapetiantz 2019[4] | baclofen | All | They detect the drug names and ADE automatically and analyse the tweets by hand then compare the ADEs detected with the ones extracted from the BNPV (called FPVD in English). They kept only the messages link to depression and a usage that is different from the usage recommended. Also, the classifiers are a bit outdated for a work done in 2018 (CRF for NE detection and SVM for relation detection…) | No | Yes | Yes | 2621 | 1866 | SRS | Frequency Type | Cases in the FPVD were significantly more informative than web forums posts for patient information (3%/6% vs 88% for the age/class of age and 46% vs 99% for the gender), treatment duration (9% vs 24%) and outcome of the ADR (1% vs 64%). But both indication and dose were more frequently retrieved in forums than in the FPVD (67% vs 24% and 27% vs 9%, respectively). Cases from web forums were significantly more frequently non-serious than the FPVD’s ones (38% vs 0.7%). Adverse events were significantly more often unexpected in forums than in the FPVD (43,8% vs 11,6%). | Forums and FPVD can be considered as 2 complementary sources of data, which confirmed what was already found in the literature. | No | No |  |
| Boeuf 2017[5] | duloxitine | All | Not reported in enough detail. Abstract only not detail of how posts were identified - 963 manually reviewed | Yes | Yes | Yes | 963 | 409 | SRS SPL/SPC | Frequency | The most frequently reported ADRs in forums were psychiatric disorders (35.3%), gastrointestinal (GI) disorders (17.9%) and general disorders and administration site conditions (13.7%). Those reported in the FPVD were nervous system disorders (16.3%), GI disorders (14.5%) and psychiatric disorders (13.1%). ADRs reported by patients in forums were less frequently serious (1.5%) than those registered in the FPVD (52.3%). Further-more, no death was identified in forums whereas 22 cases were recorded in the FPVD. We found the same proportion of unexpected ADRs between the two sources (8.1%). Fourteen unexpected ADRs reported in forums were not identified in the FPVD. Forums’ cases were less informative than those of the FPVD | Study showed a qualitative difference in the ADRs profile of duloxetine between patients’ forum posts and the FPVD. Despite less informative forums could be a complementary source of knowledge for pharmacovigilance due to the volume of data | No | No |  |
| Karapetiantz 2018[6] | baclofen | All | Not reported in enough detail. lexical match for drug names and event | No | No | No | 46,155 | 999 | SPL/SPC | Proportion | We demonstrated that applying traditional signal detection methods to web forums allows the detection of AEs described in the SPC of: on average, 60% of the SPC’s AEs were considered as signals. Only 4% of the AEs found with baclofen in forums were in its SPC. | Most adverse effects (AEs) described in the summary of product characteristics of baclofen were detected by signal detection methods. Some unexpected AEs were too. Therefore, web forums are confirmed as a complementary resource for improving current knowledge in pharmacovigilance by detecting unexpected adverse drug reactions. | No | No |  |
| Karapetiantz 2018[7] | agomelatine, baclofen, duloxetine, exenatide, strontium ranelate and tetrazepam | All | Not reported in enough detail. manual review of subset from automatic classification | No | Yes | Yes | 5149 | 3001 | SRS | Type | The cases in the FPVD were significantly more informative than posts in forums for patient description (age, sex), treatment description (dose, duration, TTO), and outcome of the ADR. But the indication for the treatment was more often found in forums. Cases were more often serious in the FPVD than in forums (46% vs. 4%), but forums more often contained an unexpected ADR than the FPVD (24% vs. 17%). Moreover, 197 unexpected ADRs identified in forums were absent from the FPVD and the distribution of the MedDRA System Organ Classes (SOCs) was different between the two data sources. | The posts were less informative (except for the indication) and focused on less serious ADRs than the FPVD cases, but more unexpected ADRs were presented in forums than in the FPVD and their SOCs were different. Thus, web forums should be considered as a secondary, but complementary source for pharmacovigilance. Also found data on outcome of AEs. | No | No |  |
| Audeh 2020[8] | agomelatine, baclofen, duloxetine, exenatide, strontium ranelate and tetrazepam | All; Named | Not reported in enough detail; This is a high-level summary paper of a series of previous works. Details about methods are reported in the lab's other works. E.g.: Vigi4Med Scraper: Audeh B, Beigbeder M, Zimmermann A, Jaillon P, Bousquet CD. “Vigi4Med Scraper: a framework for web forum structured data extraction and semantic representation. PLoS One. 2017. Manual evaluation | Yes | Yes | Yes | 5149 posts; 10,534 tweets | 1284 cases & 289 tweets | SRS DID | Type | From social media, a significant number of AEs are (1) non-serious but affect patient quality of life and (2) usually not reported by health professionals. Cases from the web forums were mostly non-serious (95.8 vs. 54.4% in the FPVD). The mean number of reactions was the same for both sources (2.3 per case in forums vs. 2.1 in the FPVD). However, if patients accurately described their experiences in web forums, they used fewer categories of ADRs, mostly attached to three main system organ classes from the Medical Dictionary for Regulatory Activities (nervous system disorders, gastrointestinal disorders, and general disorders). (3) There is a need to account for the various ways that patients mention drugs in web forums, e.g., adding lexical variations of “baclofène” (French spelling for baclofen) such as “baclo,” “Baklo,” or “Baclofen” led to the detection of 40,158 additional drug–AE couples compared with searching with only the exact match “baclofène. (4) There is no proven correlation between drug mention frequencies in social media and their prescription frequency. (5) Pregnancy topics are often discussed in web forums. More results in the tables: https://link.springer.com/article/10.1007/s40264-020-00951-2/tables/4 https://link.springer.com/article/10.1007/s40264-020-00951-2/tables/3 | (1) Vigi4Med project supports the WEB-RADR conclusions concerning the globally low quality of social media data for signal detection, but the authors think that the use of social media is inevitable and a promising method for considering the complaints and feelings of users that may not be a priority for health professionals. (2) Web forums can be a useful source to investigate ADRs that affect the patient's quality of life and medication adherence. (3) The progress in information technology and the societal need to consider patients’ experiences should motivate future research on social media surveillance for the reinforcement of classical pharmacovigilance. (4) we highlighted interest in using Twitter as a complementary resource in pharmacovigilance, the technical challenges in extracting potentially relevant data from this source (e.g., misspellings, use of abbreviations), and the difficulty of causality assessment, mainly because of restriction on the length of tweets. Also found data on quality of life and adherence. | No | No |  |
| Barakat 2022[9] | atenolol, lisinopril, amlodipine | All | Some reviews collected with term "+ the string“side effect”", SIDERs last update was in 2015 | No | No | No | 3433 | NR | SIDER | Frequency  Rank  Correlation | Many of the obtained ADRs in this study were previously known, and some of them are reported as post marketing, but still no frequencies reported for them. For example, anxiety, headache for Atenolol, and others still not reported by SIDER. Our results also showed that some known ADRs have higher similarity (frequency) than the same ADRs reported in SIDER, like Cough for Lisinopril, and Nausea for Atenolol. | A quantified, valid ranked list of most frequent ADRs have been obtained from social media | No | No |  |
| Bennett 2022[10] | COVID-19 vaccine | Named - Haematological complications—key words included thrombosis, thrombocytopenia, blood clots, haemorrhage, vaginal or menstrual bleeding, and miscarriage. | Not reported in enough detail | No | No | No | NR | 21 | SRS | Frequency | Social media, although limited by signal-to-noise considerations and an inability to verify the accuracy of patient reports of possible toxicities, provides benefits from its reach and depth. This approach provides an unparalleled mechanism to identify the first reports of potential haematology-related safety signals that might be associated with any of the numerous COVID-19 vaccines currently in use around the world. | Social media provides an opportunity to obtain initial safety reports from a vast number of the vaccinated population. | No | No |  |
| Bhattacharya 2017[11] | adalimumab (Humira), dasabuvir-ombitasvir-pari-taprevir-ritonavir (Viekira Pak), pancrelipase (Creon), le-uprolide (Lupron), ledipasvir-sofosbuvir (Harvoni), and tofacitinib (Xeljanz) | All | Not reproducible. 3rd party: epidemico | Yes | No | No | 78,289 mentions of the six products of interest: Humira (41,066), Vickira (3635), Creon (8643), Lupron (626), Harvoni (21857), Xeljanz (2462) | 3944 posts were classified as proto-AEs. Humira (3213), Vickira (37), Creon (22), Lupron, (5), Harvoni (413), Xeljanz (109) | SRS  SPL/SPC  Internal DB | Frequency  Rank | The frequency and type of PTs in social media posts were not aligned with the known distribution of MedDRA PTs aggregated at the SOC level within traditional data sources (FAERS and company database). | The value of social media proto-AEs was low in two ways: first, the data were relatively limited and not very relevant and, second, the number of posts was much lower than in traditional data sources (i.e. FAERS and company database). Also found data on patient perspectives, adherence, off label use, device and drug quality issues | No | No |  |
| Blaser 2017[12] | 163 prescription medication | Named - Nausea | Manual extraction | No | No | No | 52865 | 52865 | SPL/SPC | Frequency  Correlation | PLM nausea rates were moderately positively correlated to those reported on medication labels. (Spearman correlation: 0.59 [95% CI: 0.48, 0.68]) General trend is patient reported rates lower than those on labels | In general, self-reported rates of nausea associated with medication use were lower than those reported in medication labels. Although considered a definitive resource for medication information, this discrepancy demonstrates that medication labels may not comprehensively describe the patient experience. Also found data on patient perspectives | Yes | No |  |
| Borchert 2019[13] | eszopiclone, zaleplon, zolpidem, rameelteon, suvoreexant | All |  | No | No | No | 1407 reviews: eszopiclone (n=239), ramelteon (n=72),suvorexant (n=324), zaleplon (n=82), or zolpidem (n=690) | 2030: eszopiclone (n=319), ramelteon (n=104),suvorexant (n=567), zaleplon (n=82), or zolpidem (n=958) | SRS | Type  Frequency  Rank | AEs found consistent with what is reported in FAERS though the rank order may differ | our results show that online reviews are guided primarily by considerations of drug efficacy and tolerability and match well with adverse event data reported to FAERS, we conclude that patient online reviews offer a valuable supplement to traditional adverse event reporting systems. Also found data on patient perspectives, quality of life, cost efficacy, functional benefits. | No | No |  |
| BrattigCorreia 2019[14] | fluoxetine, sertraline, paroxetine, citalopram, trazodone, escitalopram, fluvoxamine, carbamazepine, clobazam, diazepam, lacosamide, lamotrigine, levetiracetam, oxcarbazepine, seizure meds, fentanyl and oxycodone | All | Dictionary match for detection, no validation discussed | No | No | No | 966,437 (Twitter), 2,439,964 (Instagram) | NR | SIDER | Type | According to our gold standards, it contains 9 known ADR inthe top 25 proximity relations—between D-MT pairs only | Thus, social media in general, and in special Instagram data, shows great potential for public health monitoring and surveillance for DDI and ADR, as it can add precision in public health studies. Preliminary analysis suggests that metric edges in general are not very good predictors of DDI or ADR. However, when restricting analysis only to terms used to extract user timelines, we discovered that a large proportion of metric edges in these subnetworks are of known DDI or ADR. Also learned that social media may play an increasingly important role in the investigation of ADR, specifically from DDI, specially as longer historical social media data becomes available. We found anecdotal evidence that mentions of DDIin social media may actually precede that of official means, such as scientific publications | No | No |  |
| Campillos-Llanos 2019[15] | baclofen, levothyroxine and vaccine | All | Manual annotation review | No | No | No | 41,551 | 1160 | SPL/SPC | Type | The expert identified 68 undocumented ADRs (22.6% of selected items; 28 need more context to be confirmed). Unknown ADRs mostly involved levothyroxine (52 cases), baclofen (6), Engerix® (2), Infanrix® (2), Pentavac® (3), Priorix® (1) and Repevax® (2). | entities not found in SCPs might express drug misuse or unknown ADRs | Yes | No |  |
| vanStekelenborg 2019[16] | 75 medications | All | 3rd party: Epidemico. Used lexical matching and LR to find medical event reports in posts with low recall. Poor performance: the WEB_RADR AE recognition algorithm had a recall of 0.39 and a precision of 0.70 for classifying posts containing an AE and a recall of 0.20 and a precision of 0.38 for extraction | No | No | No | 4,294,658 | 465,608 | SPL/SPC  Reference Standard | Timing | using these social media as a broad-based stand-alone data source for statistical SD in pharmacovigilance yielded no predictive ability in two complementary reference sets of signals and label changes. | WEB-RADR does not recommend the use of general social media, as exemplified by Facebook and Twitter, for broad statistical SD. However, there may be added value derived from social media channels for specific niche areas such as those seen in the case studies related to drug abuse and pregnancy-related outcomes. Subject to further research, primarily to enhance AE recognition algorithms, the scope and utility of social media may broaden over time. Also found insights from drug abuse or pregnancy related outcomes. | Yes | No |  |
| Caster 2018[17] | 75 medications | All | 3rd party: Epidemico. Used lexical matching and LR to find medical event reports in posts with low recall. Poor performance: the WEB_RADR AE recognition algorithm had a recall of 0.39 and a precision of 0.70 for classifying posts containing an AE and a recall of 0.20 and a precision of 0.38 for extraction | No | No | No | 4,294,658 | 465,608 | SRS | Frequency  Timing  ROC | Across all algorithms, the area under the receiver operating characteristic curve for Twitter/Facebook varied between 0.47 and 0.53 for the WEB-RADR reference set and between 0.48 and 0.53 for the Harpaz reference set. For VigiBase, the ranges were 0.64–0.69 and 0.55–0.67, respectively. In Twitter/Facebook, at best, 31 (16%) and four (6%) positive controls were detected prior to their index dates in the WEB-RADR and Harpaz references, respectively. In VigiBase, the correspond-ing numbers were 66 (33%) and 17 (27%). In Social 0.7, there were in total five (3%) PECs detected strictly before their index dates, with any disproportionality algorithm. The corresponding numbers were 31 (16%) and 1 (0.5%) for Social 0.4 and patient forum posts, respectively, while in VigiBase there were 66 (33%) such PECs. | results clearly suggest that broad-ranging statistical signal detection in Twitter and Facebook, using currently available methods for adverse event recognition, performs poorly and cannot be recommended at the expense of other pharmacovigilance activities | No | No |  |
| Chen 2018[18] | methylphenidate | All | Rule based lexical match; automatic identification required drug/AE mention in the same sentence | No | Yes | No | 3443 | 61 | SRS SPL  SPC | Type  Proportion | 66.7% of the identified relationships (26 of 39) are detected as a signal, among which,38.5% (10 of 26) have been mentioned in the product SPC and 88.5% (23 of 26) have been alarmed in VigiBase. | This work emphasizes the potential interest of monitoring adverse drug reactions on social media but there is still insufficient evidence to define how such monitoring should be integrated within the current pharmacovigilance process. Also found data on patient perspectives, off label use and abuse | No | No |  |
| DeLangen 2017[19] | birth control arm implants | All | Not reported in enough detail. Abstract only - not details of methods | No | No | No | NR | 1316 | Literature | Type | Mood conditions or emotional states were only found in social media and represented the majority of the 180 suspected ARs of the psychiatric disorders SOC. Unsurprisingly social media ARs included vernacular reported terms and lacked completeness. Unexpected serious cases were found only in literature with two cases of pseudotumor cerebri. | Reports from BCAI users in social media and published scientific literature complemented each other to provide a comprehensive overview of ARs to BCAIs. Scientific literature remains the preferred source for unexpected serious ARs. Social media is a better source to identify emotional- and social-related ARs. Also found data on mood and emotional states. | No | No |  |
| denHollander 2022[20] | imatinib | All | Not reported in enough detail. No details on algorithm; performance 30.5% FP, normalizer64% | No | No | No | 125,161 | NR | Trials  Survey | Frequency  Type  Rank | Fatigue and muscle pain or cramps were reported most frequently. Seven out of 10 most reported symptoms (i.e. fatigue, muscle pain or cramps, facial swelling, joint pain, skin problems, diarrhoea, and oedema) overlapped between the two sources. Alopecia was frequently mentioned on the forum, but not in the survey. Four out of 10 most reported symptoms on the online forum are covered by the EORTC QLQ-C30. The EORTC-SBQ and EORTC Item Library cover 9 and 10 symptoms, respectively | This first overview of patient-reported imatinib-related symptoms from two data sources helps to determine coverage of items in existing questionnaires and prioritize HRQoL issues. Combining cancer-generic instruments with treatment-specific item lists will improve future HRQoL assessment in care and research in GIST patients using TKI. Useful data on quality of life | Yes | Yes |  |
| Dirkson 2022[21] | imatinib, sunitinib, regorafenib, avapritinib, ripretinib, nilotinib, pazopanib, ponatinib, sorafenib | All |  | No | Yes | Yes | 121,561 | 21,051 | SPL/SPC  Trials | Frequency | Forum data can also indicate ADEs that are novel for all imatinib users. Thin skin, clouded consciousness, menopausal flushing, change in hair color, and tooth problems are examples of adverse drug events found on the forum that were not reported in either registration trials for GIST or in the general SmPC. | we have shown with a case study of an online forum for GIST patients that patient forums can pro-vide real-world data for both long-term ADEs, such as osteoporosis and tremors for imatinib, as well as for ADEs that were not found in the original registration trials, such as dry eyes and muscle cramping for imatinib. Also found data on patient perspectives and quality of life. | Yes | Yes |  |
| DeRosa 2021[22] | dutasteride, finasteride, minoxidil | All | 3rd Party:dbPedia Spotlight | No | No | No | 20,560 | NR | Literature | Correlation | with a residual thresholdof±4%, 91% of drug and side effect correlations extracted from tweets can be considered reliable. | The results of the proposed research are a starting point that supports the consideration according to which cross-relating different sources of information allows to measure the quality of shared information as a countermeasure for misinformation and fake news | No | Yes |  |
| Dreyfus 2017[23] | 3 drugs | Named - Seven AEs | 3rd Party: epidemico. abstract; Proto-AEs (social media posts with resemblance to AEs)were provided by Epidemico using MedWatcher So-cial, a platform that classifies data using machinelearning | No | No | No | NR | NR | SRS  SPL/SPC  Claims | Predictive Value | The sensitivity of social media, administrative claims and FAERS to identify listed AEs was4.8% (95% CI 3.5%, 6.6%), 1.3% (0.6%,2.4%) and15.9% (13.5%,18.7%), respectively. Specificity was highest with social media at 67.9% versus 59.4% and11.6% for claims and FAERS respectively. PPV was highest with social media at 67.9% versus 27.8% and30.3% for claims and FAERS respectively. NPV was similar for all three sources, ranging from 4.8% to5.4%. Of the seven drug-events, five were correctly identified by social media and FAERs using the disproportionality score, while four were identified by claims data | Social media performed as well (FAERS)or bet-ter than the other data sources (administrative claims) at identifying events associated with a drug, using the label as the gold standard. | No | No |  |
| Eslami 2020[24] | neurotic drugs, anti-pregnancy drugs and digestion drugs | All | Not reported in enough detail. Limited discussion on training, extraction of side effects from comparitors, no validation that mention was an AE and not the indication or other symptom mention | No | No | No | 13,073 | NR | SIDER Online Medical Site | Frequency Type | users’ comments were different from the side effects of drugs reported in the web-sites Sider and WebMD in case of the three classes of drugs; however, the websites had reported some side effects but with a low frequency. | The results show that the data from social media may have noise, or may not be reliable. Accordingly, social media can be employed as a secondary source in identifying the side effects of drugs rather than a substitution for traditional and scientific methods of identifying side effects. The option of reporting ‘unregistered side effect’ shows that a great deal of data, which have not been reported in drug studies, can be extracted from social media. Also found data on patient perspectives. | No | No |  |
| Farooq 2020[25] | alprazolam, Adderall, fluoxetine, venlafaxine, adalimumab,lamotrigine, quetiapine, trazodone, paroxetine, metronidazole and miconazole | All | manually annotated/classified health v non-health tweets, no mention of manual validation of AE mentions, used lexicon match | No | No | Yes | 89,136 tweets (1.6 M collected) | 599 (based on unique ADRs found for each drug, no details given) | SRS Online Medical Site | Type | (1) on average 43 known ADRs are shared between Twitter and FAERS datasets; (2) able to recover on average 7 known side effects from Twitter data that are not reported on FAERS; (3) a high concordance with FAERS, Medeffect and Drugs.com; (4) able to manually validate some of the under-reported side effect predicted by their model using literature search. | The data available on Twitter is without any specific medical focus and suffers from high false positives. Such false positives can be removed by our methodology (filtering tweets with a lexicon and removing false positives using a classifier). The approach could highlight possible under-reported ADRs, however, subsequent manual examination by experts is required to confirm these ADRs. The unknown side effects found by our model are the possible under-reported ADRs that were not present in the list of known ADRs and need further clinical validation. | Yes | No |  |
| Ferawati 2022[26] | COVID-19 vaccines | All; Named - side effects, tiredness, headache, muscle pain, chills, fever, nausea | | No | No | No | 286,887 Japanese tweets and 14,484 Indonesian tweets | 14,259 | Trials | Frequency | We observed some differences in the ratio of side effects between the public reports and tweets. Specifically, fever was mentioned much more frequently in tweets than would be expected based on the public reports. We also observed differences in side effects reported between Pfizer and Moderna vaccines from Japan and Indonesia, with more side effects reported for the Pfizer vaccine in Japanese tweets and more side effects with the Moderna vaccine reported in Indonesian tweets. | we present cautious optimism that Twitter can prove useful for infodemiological surveillance for vaccine side effects that is best suited for detecting prevalences of fever symptoms in Japanese and Indonesian populations | Yes | No | |
| Gavrielov-Yusim 2019[27] | All | All |  | No | No | Yes | NR | 2500 | SRS | Frequency | We found that co‐occurrence and NLP produce AEs, which are 57% and93% consistent with VigiBase AEs, respectively. Among the SDRs identified both in SM and in VigiBase, up to 55.3% were identified earlier in co‐occurrence, and up to32.1% were identified earlier in NLP‐processed SM. Using lift in SM SD provided performance similar to frequentist methods, both in co‐occurrence and in NLP‐processed AEs | When comparing SM AEs with spontaneous reporting system AEs, it was demonstrated that a large fraction of SM AEs was also found in VigiBase. The level of SM and VigiBase concordance implies that (a)patient forums raise AEs, which are consistent with spontaneously reported AEs, and (b) adding SM to routine pharmacovigilance will likely add a manageable amount of complementary information. Moreover, both SM processing methods were able to identify SDRs prior to VigiBase, with up to 55.3% of early SDR detection in co‐occurrence and up to 32.1% of early SDR detection in NLP. Our results demonstrated that NLP‐processed SM is more concordant with spontaneous reports, both in AE content and in SDR detection time, than the co‐occurrence data. Our results indicate that using SM as a data source complementary to traditional pharmacovigilance sources should be considered further. Also found data on quality of life. | No | No |  |
| Golder 2021[28] | atorvastatin, fluvastatin, pravastatin, rosuvastatin, simvastatin, lovastatin, pitavastatin, cerivastatin | All |  | No | Yes | Yes | 12,649 tweets | 356 | SRS Literature | Frequency Rank Proportion | Compared with other sources, patients on social media are proportionally far more likely to complain about musculoskeletal symptoms than other adverse events. Most adverse events showed a high level of agreement between Twitter and regulatory data. DIDs tend to demonstrate similar patterns but not as strongly. Systematic reviews tend to examine pre-specified adverse events or those reported by trial investigators | Combining the data from multiple sources, albeit challenging, may provide a broader safety profile of any medication. Systematically collected social media reports may be able to contribute information on the most pertinent adverse effects to patients. Also found data on patient perspectives. | Yes | No |  |
| Han 2020[29] | oseltamivir, peramivir, zanamivir, and laninamivir | All | manual extraction | No | No | No | 396 review comments and 440 subjects from 413 reviewers | 575 | SRS | Frequency | Psychiatric disorders were most common in younger and older patients, while gastrointestinal disorders were most common in adult given oseltamivir in the WebMD. In FAERS Psychiatric disorders (1880, 12.20%) were the most common AE clinical symptoms for oseltamivir. The occurrence rates of psychiatric disorders of zanamivir, laninamivir, and peramivir were 9.09%, 2.38%, and 1.16%, respectively. Cardiac and vascular disorders (9.57% and 9.28%, respectively) were the most common AEs for peramivir, while general disorders and administration site conditions (113, 12.68%) were the most common AEs for zanamivir | However, in the WebMD data, we could not detect signals by these disproportionality analyses due to the small number of AE cases, although psychiatric and gastrointestinal disorders were the most common AEs reported. | Yes | No |  |
| Harpster 2018[30] | ciprofloxin, levofloxacin | All | Not reported in enough detail. Abstract - no description on AE extraction | No | No | No | 600 | 202 | SRS | Frequency Type | The top six Twitter-reported ADRs were nausea (14.1%), musculoskeletal disorders (14.1%), pain (12.1%), drug hypersensitivity (10.1%), taste disturbances (7.1%), and mental disorders (6.1%). The top six FAERS-reported ADRs were peripheral neuropathy (46.0%), drug hypersensitivity (9.3%), mental disorder (9.0%), musculoskeletal disorders (6.9%), central nervous system disorders (4.3%) and cardiovascular disorders (4.3%). Out of 300 levofloxacin tweets, 103 (34.3%) contained reported ADRs. The top six Twitter-reported ADRs were musculoskeletal disorder (19.4%), treatment failure (14.6%), mental disorders (11.7%),pain (10.7%), drug hypersensitivity (7.8%), and insomnia(7.8%). The top six FAERS-reported ADRs were musculoskeletal disorders (36.2%), peripheral neuropathy (31.1%), mental disorders (9.9%), drug hypersensitivity (5.4%), pain (4.7%) and pain in extremity (2.8%) | The top six ADRs for Twitter compared to FAERS had many differences for both ciprofloxacin and levofloxacin. The differences in frequencies of ADRs could be due to what patient’s report to their healthcare providers versus to their Twitter followers. Twitter and other social media platforms could potentially serve as another method to collect ADR data | No | No |  |
| Hoang 2018[31] | ibuprofen (Advil, Motrin), lisinopril (Prinivil, Zestril), ramipril (Altace), perindopril (Aceon), captopril (Capoten), quetiapine (Seroquel), olanzapine (Zyprexa), sertraline (Zoloft), paroxetine (Paxil), citalopram (Celexa), lamotrigine (Lamictal), pregabalin (Lyrica), warfarin (Counmadin), rivaroxaban (Xarelto), dabigatran (Pradaxa), rosuvastatin (Crestor), atorvastatin (Lipitor), simvastatin (Zocor) | Named; All | The ground truth and human validation could be explained more specifically. Lexical match for identifying drug and AE entities - search for data using drug-ae terms | No | Yes | Yes | 1,191,767 tweets | 8089 | SIDER | Type | AC-SPASM (their AE detection model) recognizes authentic posts with F1 – the harmonic mean of precision and recall of 80%, and estimates user credibility with precision@10 = 90% and NDCG@10 – a measure for top-10 ranking quality of 96%. Upon validation against known ADEs, AC-SPASM achieves F1 = 91%, outperforming state-of-the-art baseline models by 32% (p < 0.05). Also, AC-SPASM obtains precision@456 = 73% and NDCG@456 = 94% in detecting and prioritizing unknown potential ADE signals for further investigation. Furthermore, the results show that AC-SPASM is scalable to large datasets. | Social media is a promising open data source for the timely signalling of potential ADEs. Taking into account the content authenticity and user credibility improves the detection of ADEs from social media. Our work generates hypotheses to reduce experts’ guesswork in identifying unknown potential ADEs. | Yes | Yes |  |
| Hussain 2022[32] | COVID-19 vaccine | Named - AEFI search terms: 1.Allergy OR allergic OR anaphylaxis OR Eczema OR itch OR rash [Allergy, combine with 18] 2. Arrhythmia OR palpitations OR tach (Combine with 8, under Cardiac-related) 3.Asthma, 4.Ataxia, [Neurological, combine with 5, 15, 16, 27] 5.Bell’s palsy 6.Breathless OR breath OR sinus OR nasal OR dyspnoea OR throat OR mouth 7.Cough 8.cardiac OR heart OR cardio OR chest 9. Ear pain OR vertigo OR tinnitus OR dizz OR balance 10. Diarrhea OR loose stool (Gastrointestinal, with 11, 12, 13 11. Abdo 12. Gastritis OR duodenitis OR reflux OR gord 13.Appetite 14.Fever OR chills OR chilly OR pyrexia OR sweat OR temp OR flush OR febrile [Systemic, combine with 21, 22] 15. GBS OR Guillain-Barre, 16. Headache OR head pain OR migraine 17. (Injection OR site) AND (pain OR red OR swell OR bruis OR urticaria) [Injection Site] 18. Skin AND (pain OR red OR swell OR bruis OR urticaria) 19. Pain, 20. Sleep OR dream OR anxiety OR anxious OR panic OR psych OR mood OR depress OR insomnia OR mental OR sad OR unhappy 21. Lethargy OR malaise OR fatigue OR tired, 22. Muscle AND (ache OR pain) OR myalgia [Combine with 31] 23. Lymph, 24. Nasal congestion OR block nose, 25. Swelling 26. Eye AND (pain OR red OR swell OR allergy OR itch OR irrit), Optic neuritis OR photophobia OR blind OR blur 27. Paresthesia OR tingl OR tremor OR seizure 28. Flu OR influenza 29. Vomit (combine with 30) 30. Nausea 31. Joint pain OR arthralgia 32. stroke, ITP, thrombocytopenia, clot, low platelets, thrombosis, CVST, DVT, PE, haemorrhage, embolism, infarct | lexical match for specific AE terms | No | No | No | 46,762 unique Facebook posts and 74,644 tweets | Allergy OR allergic OR anaphylaxis OR Eczema OR itch OR rash 53924 9% Arrhythmia OR palpitations OR tach 14821 3% Asthma 12936 2% Ataxia 17606 3% Bell’s palsy 11909 2% Breathless OR breath OR sinus OR nasal OR dyspnoea OR throat OR mouth 14287 2% Cough 21752 4% cardiac OR heart OR cardio OR chest 18819 3% Ear pain OR vertigo OR tinnitus OR dizz OR balance 18421 3% Diarrhea OR loose stool 16559 3% Abdo 16574 3% Gastritis OR duodenitis OR reflux OR gord 11569 2% Appetite 79132 14% Fever OR chills OR chilly OR pyrexia OR sweat OR temp OR flush OR febrile 12707 2% GBS OR Guillain-Barre, 9576 2% Headache OR head pain OR migraine 10641 2% (Injection OR site) AND (pain OR red OR swell OR bruis OR urticaria) 56152 10% Skin AND (pain OR red OR swell OR bruis OR urticaria) 7314 1% Pain, 11006 2% Sleep OR dream OR anxiety OR anxious OR panic OR psych OR mood OR depress OR insomnia OR mental 9241 2% Lethargy OR malaise OR fatigue OR tired, 22829 4% Muscle AND (ache OR pain) OR myalgia 6849 1% Lymph, 11331 2% Nasal congestion OR block nose, 8567 1% Swelling 9617 2% Eye AND (pain OR red OR swell OR allergy OR itch OR irrit), Optic neuritis OR photophobia OR blind OR blur 20070 3% Paresthesia OR tingl OR tremor OR seizure 8115 1% Flu OR influenza 12109 2% Vomit, 4401 1% Nausea, 3232 1% Joint pain OR arthralgia 7028 1% stroke, ITP, thrombocytopenia, clot, low platelet, thrombosis, CVST, DVT, PE, haemorrhage, embolism, infarct 43907 8% | SRS | Frequency | The most frequently mentioned symptoms were broadly found to be similar to those most frequently reported in the YellowCard system [12]. For example, the most commonly reported AEFI on Yellow Card was related to injection-site reactions and generalized symptoms (e.g., fever, headache, lethargy, muscle ache, flu, vomit, nausea), which in our analysis accounted for10% and 13% of the mentions, respectively. | Overall, this work has confirmed the opportunity for social media analysis to provide insights into public sentiments and complement more established pharmacovigilance efforts. It is important to note that we did not aim to identify new side-effects; our objective was rather to monitor trends relating to currently reported ones | Yes | Yes |  |
| Jarynowski 2021[33] | COVID-19 vaccine | All |  | No | No | No | 11,515 | 11,515 | SRS Trials | Frequency Type | Telegram users reported significantly more (5.6-fold on average) systemic AEs than their peers, according to the results of thephase 3 clinical trials published in The Lancet. However, the AEs reported in Telegram posts were consistent (Pearson correlation r=0.94, P=.02) with those reported in the Argentinian post marketing AE registry | After the Sputnik V vaccination, Russian Telegram users reported mostly pain, fever, and fatigue. The SputnikV AE profile was comparable with other vector COVID-19 vaccines. Discussion on social media could provide meaningful information about the AE profile of novel vaccines. | No | No |  |
| Jiang 2020[34] | buprenorphine, fentanyl, hydrocodone, oxycodone | Named; All |  | Yes | Yes | No | 3.6 million tweets | 624 | SRS Literature Trials SPL/SPC | Frequency | We inferred a total of 765 opioid effect relations, among which 624 are SE (side effect) relations and 141 are IND (beneficial) relations, as shown in Table 1 which lists the results of initial screening directly from the inference of relation similarity. The results came from verification with the SIDER data. Among all the 765 inferred relations, 250 (= 218 SEs + 32 INDs) are known, matching those in the SIDER database, and 515 (= 406 SEs + 109 INDs) are potential, meaning that they were not found in SIDER and may or may have not been reported. | Their method has the power and utility to detect opioid-related AEs from social media. "It is conceivable that our approach can be applied to discovery of potentially unreported effects of other medications." | No | No |  |
| KhademiHabibabadi 2023[35] | COVID-19 vaccine | Named - ache, fatigue, lethargy, tired, weakness, pain, sore, hurt, aches, aching, chills, fever,nausea, drowsy, confused, dizziness,“light head,”unsteady, dizzy, reaction, syncope, faint,muscle, myalgia, joint, arthralgia, chest, myocarditis, myorcarditis, pericarditis, rash, hives,diarrhea,diarrhea,paresthesia,tingl,burning,vomiting,swollen,inflamed, cough,flush,itchy, scratchy, redness, clot, bleed, shaking,“shortnessofbreath,”“bad taste,”smell,abdominal, stomach, seizure,fit, numbness, drooping, allergic, anaphylaxis, period | | No | No | Yes | 2,901,096 | 1,021,434 with personal health mention | SRS | Rank Proportion | We identified a correlation between the proportions ofCOVID-19 vaccine reactions in Twitter posts containing vaccine-related PHMs and SAEFVIC AEFI surveillance reports. Common and expected reactions in the top six topics generally match between the data sets, although usually in a different order. There is significant reporting about bleeding issues in the Twitter data, especially in association with Vaxzevria, but also in the other vaccine brands. Although some of this is due to the model inaccurately identifying conversations that are not actually PHM of vaccine reactions, a manual examination of the data showed that genuine bleeding reports are considerably large proportion, compared with SAEFVIC reporting. On the other hand, SAEFVIC had a greater proportion of heart-related reports compared with Twitter. | Our study has provided an initial validation step suggesting that detection of PHMs on Twitter can be used to enhance vaccine safety surveillance to improve timely responses to emerging vaccine events. Social media also provides insights into how people are experiencing the pandemic and vaccine rollout. Also found data on patient perspectives. | No | No | |
| Kim 2020[36] | Adderall, Dexedrine, Ritalin, Concerta, Focalin, Daytrana, Metadate, Methylin, Vyvanse, Clonidine and Guafacine | All; only AE in 2 to 5 years old children. | ADR lexicon built using SIDER/CHV wiki, co-occurence frequency to id drug-ae pairs | No | No | No | 3,504 posts and their corresponding 20,240 responses/comments, which were reduced to 965 after filtering by age, drug, and ADRs (for focusing on the specific disease and population). | 7 types, with no frequencies reported | SPL/SPC Literature | Other | Our study identified four associations supported by FDA product labels. Among them, two ADRs are found in section 6.1 clinical trial experience of the drug labels: “Rash” is labelled as an adverse reaction of Clonidine and “anorexia” of Concerta based on trial findings. The other two drug-ADR associations, Clonidine=> hallucination and Focalin=> agitation, have been previously reported in section 6.2 post-marketing experience of the FDA product labels. Three neuropsychiatric ADRs (mental disorder, mood swings, and panic attack) were found to be associated only with stimulants. Interestingly, all three types of medications of stimulants (Amphetamine, Methylphenidate, and Lisdexamfetamine) showed these signals. | There is great potential to identify adverse drug reactions from consumer-generated content. Consumers’ activities of sharing their experiences in taking drugs and the drug effects make it a promising resource to identify ADRs. Preschool-aged children usually cannot participate in clinical trials, so health consumer-generated content provides a unique opportunity for the academy and pharmaceutical industry.  . | No | No |  |
| Koutkias 2017[37] | haloperidol, clozapine, apixaba | Named - myocarditis, cardiomyopathy, cerebral hemorrhage | Lexical match to identify drug-event pairs | No | No | No | NR | 75 | SRS Literature | Type | Out of the 56 Twitter posts obtained, 33 were categorized as fully relevant (referring at published studies, clozapine labelling warning changes, news about the ADR, as well as personal experiences), 10 as partially relevant (parts of a relevant discussion thread), and 13 were excluded (from which 9messages were part of a discussion thread about the genetic mechanism of clozapine). The first, fully relevant Twitter post was made available on March 2010. Interestingly, among the fully relevant Twitter posts, we discriminated three posts (two generated from the same user) corresponding to potential patient experiences of the ADR, and two potential healthcare professional experiences regarding this ADR. | The analysis of the obtained data provided interesting insights (identification of potential patient and health-care professional experiences regarding ADRs in Twitter, information/arguments against an ADRexistence across all sources), while illustrating the benefits (complementing data from multiple sources to strengthen/confirm evidence) and the underlying challenges (selecting search terms, data presentation) of exploiting heterogeneous information sources, thereby advocating the need for the proposed framework. This work contributes in establishing a continuous learning system for drug safety surveillance by exploiting heterogeneous publicly available data sources via appropriate support tools. Also found data on healthcare professional experiences. | No | No |  |
| Kurzinger 2018[38] | insulin glargine and zolpidem | All | Not reported in enough detail. Didn't specify the volume of SM text analysed.. | No | No | No | NR | 57 for insulin glargine and 86 for zolpidem. | SRS | Type  Timing Proportion | The numbers of drug‐event pairs identified in patient's forums over the 2005‐2015 period for the two products were 57 and 86, respectively. Comparison analysis showed that the sensitivity ranged from 55 to 75.4%, the specificity from 31.7 to 84%, the PPV from 50 to 68.3%, the NPV from 47.6 to 80.4%, and the accuracy from 53 to 72.7%. The AUC reached 0.73 when using the metric EBGM. Up to 32.1% of the SDRs were detected earlier in the patient's forums than in VigiBase®. | The overall performance of data from web patient's medical forums was good, and it may be a valuable source for signal detection. In total, up to 32.1% of the SDRs could have been detected earlier, thus facilitating increased safety of the patients. | No | No |  |
| Kurzinger 2018[39] | insulin glargine and zolpidem teriflunomide. | All |  | No | Yes | Yes | 9256 | 545 drug-event pairs (33 unique drug-event pairs for teriflunomide, 194 for insulin glargine, and318 for zolpidem) | SRS | Type  Timing Proportion | The comparison analysis showed that the sensitivity ranged from 29% to 50.6%, the specificity from 86.1% to 95.5%,the PPV from 51.2% to 75.4%, the NPV from 68.5% to 91.6%, and the accuracy from 68% to 87.7%. The AUC reached 0.85when using the metric empirical Bayes geometric mean. Up to 38% (12/32) of the SDRs were detected earlier in the forums than that in VigiBase. Among 545 drug-event pairs from the forums, only 123 were not identified in VigiBase; those 123 drug-event pairs corresponded each to only one message in the forums. | The overall performance was good, showing that data from medical forums may be a valuable source for signal detection. In total, up to 38% (12/32) of the SDRs could have been detected earlier, thus, ensuring the increased safety of patients. | No | No |  |
| Lardon 2018[40] | 33 drugs | All | manual review | No | No | Yes | 10,534 | 289 case reports | SRS SPL/SPC DID | Frequency Type | Overall, 20 of the 289 reportable ADRs were considered to beunexpected, because they were not documented in the corresponding French SPC available during the study period. One was reported in the Drugdex database, 4 were reported in the Martindale database and fourunique relationships between a drug and an event were reported at least once in the French national pharmacovigilance database (BNPV, the French acronym for Base National ADE PharmacoVigilance), corresponding to eight case reports. Finally, 9 of the 289 ADRs were totally and fully unexpected, because never mentioned or reported in any source. | With the use of dedicated tools, Twitter could become a complementary source of information for pharmacovigilance, despite a major limitation regarding causality assessment of ADRs in individual tweets, which may improve with the new limitation to 280 characters per tweet. | No | No |  |
| Lebanova 2019[41] | omeprazole and famotidine | All | abstract; Manual review for AE | No | No | No | 610 (omeprazole), 321 (Famotidine) | 11 (omeprazole), 3 (Famotidine) | SPL/SPC | Frequency | No unknown and new ADRs were identified for both products. | Online discussion forums are easily accessible potential source of ADR reports. They could be used for signal detection and confirmation. User anonymity and potential for multiple reports of a single ADR event are among the main challenges. | No | No |  |
| Lee 2023[42] | metoclopramide, chlorpheniramine, and ketoprofen | All | lexical match | No | No | No | 25,693 posts 14,156 crawled posts on ketoprofen | 828 posts containing a word in the side effects dictionary were extracted at least once | SRS | Rank | The SOC rankings of the 960 patients (2013–2017) in KAERS reporting ADRs with ketoprofen followed a similar pattern (Fig. 6). Spontaneously reported KAERS-based and detected SNS-based adverse events resembled the top SOC groups. | Based on the drug information alone, it was confirmed that drug side effects may be monitored according to the SNS data and from the perspective of consumers. Thus, SNS data can also be used to search for ADR information and identify the characteristics of patients presenting with ADR. | No | No |  |
| Li 2019[43] | glucophage (Metformin) | All |  | No | No | No | 1562 comments | 870 | SRS | Rank | confirms the findings of FDA reports, showing all these ADRs come from the top 10 list of FDA reports. In our analysis, we found diarrhea to be the top ADR when taking Glucophage. However, the top ADRs in FDA reports, such as blood glucose increase, and weight loss were not reported by many patients in AskaPatient.com. | Our case study on Glucophage demonstrates the usefulness of the framework and reveals patterns which will help answer important questions in the domain of knowledge discovery in online health communities. This framework can be also applied to any medicine to discover its side effects, association and patterns, and even prediction. | No | No |  |
| Li 2020[44] | Drugs in RxNORM | All | some described in previous paper | No | Yes | No | 18.9 million | 192,000 tweets | SRS SIDER | Type proportion | Our study suggests that Twitter postings of drug-related AEs tend to focus on much fewer AEs (about 80) than in FAERS (about 15,000) and less-serious AEs, such as dizziness, pain, and nausea, which affect quality of life rather than being clinically serious and significant AEs. | The accuracy of signal detection using social media can be improved by combining signals with those from SRSs. However, the combined system cannot achieve better AUC performance than data from FAERS alone, which may indicate that Twitter data are not ready to be integrated into a purely data-driven combination system | Yes | No |  |
| Lian 2022[45] | COVID-19 vaccine | All |  | No | Yes | Yes | 111,229 | 66,499 | SRS | Frequency Rank | Sore to touch, fatigue, and headaches were the top three most common adverse events for all three vaccines. All these top events appeared to be mild symptoms. In comparing our results to the data reported in the Vaccine Adverse Event Reporting System, we found that “sore to touch” was the most common VAE identified through Twitter data but did not show up in the VAERS. This is probably because “sore to touch“ is such a minor yet common adverse event that individuals do not bother to report it to physicians and through the governmental portal, but they nevertheless talk about it in describing their vaccine experiences on Twitter. This discrepancy shows the relative strength and weakness of identifying VAEs through social media content that people share informally and through more formal report portals operated by the government and pharmaceutical companies. | Our findings demonstrate the feasibility of using social media data to monitor VAEs. To the best of our knowledge, this is the first study to identify COVID-19 vaccine adverse event signals from social media. It can be an excellent supplement to the existing vaccine pharmacovigilance systems. Also found data on patient perspectives. | No | No |  |
| Liu 2017[46] | diabetes and cardiovascular related | All |  | No | No | Yes | 1,072,474 posts; 2nd Experiment: 305,169 (twitter/ADA forum) | 1,297 (beta blocker test bed); 628 Experiment 2 | SRS SIDER | Frequency Timing | FAERS focuses on severe ADEs, such as “loss of consciousness” and “death,” while forum reports concentrate on mild ADEs such as “anxiety” and “dizziness.” Forums seem to be more symptom derived describers while FAERS seems to be more diagnosis derived describers. The healthcare professionals might describe palpitations, fast heart rate, and arrhythmia from forum discussions as atrial fibrillation. By January2015, we have obtained 407 reports related to Afrezza from Twitter. Among them, 127 contained drug indications or adverse events. Meanwhile, there is only one case report about Afrezza in FAERS database. | Based on the results of adverse drug event extraction, we are able to confirm the strengths and weaknesses of different social media data sources. Twitter data is sensitive to emerging drug safety issues. We can obtain adverse events for new drugs ahead of FDA from Twitter. However, there are a large amount of repeated reports for well-established events, limiting the ability of Twitter to establish prevalence of the events. Forum discussions can provide insight into long-term effects of treatments on patients. They are more reliable and less biased toward well-known serious adverse drug events. | No | No |  |
| MacKinlay 2017[47] | 3050 | All | Lexical/synonym matching to find AEs; classifier trained had low P/R/F1 | No | Yes | No | 230 million tweets | NR | SRS | Type | Largest discrepancies were in FN (I.e., Yes in FAERS but not detected in Twitter), however they also detected AEs mentioned in Twitter not in FAERS (e.g., inexact mentions such as feel sick, or potential unknown such as stomachache or pyrosis for certain drug) | We suspect the ADRs being uncovered using Twitter are at least somewhat complementary to those in FAERS (out of all our false positives, it is likely that some are actually novel ADRs), but this is difficult to assess without further investigation. | No | No |  |
| Maskell 2017[48] | NR | All | Third Party: Epidemico. Abstract only | No | No | No | 194,496 | NR | SRS | Frequency Type | The total number of high-quality social media posts is approximately 10% of the number of spontaneous reports in the same time period. For some PTs (e.g., “cardiac arrest”) , the estimated rate of reporting is substantially lower (<0.1%). Despite this, there are certain PTs (e.g., “altered state of consciousness”) for which the estimated underlying reporting rate is substantially higher in social media than in spontaneous reporting. | Social media can provide information that complements that stored in spontaneous reporting databases. This implies that there is a need to develop algorithms for combining the information present in the two sources | No | No |  |
| Matsuda 2017[49] | depressive disorders and rheumatoid arthritis | All |  | Yes | Yes | No | NR | 14,355 | SRS | Frequency Type  Rank | Comparison of medical terms observed in tōbyōkiblogs with those in an external ADR reporting database showed that subjective and symptomatic events and general terms tended to be frequently observed in tōbyōkiblogs (e.g., anxiety, headache, and pain), whereas events using more technical medical terms (e.g., syndrome and abnormal laboratory test result) tended to be observed frequently in the ADR database | Pharmacovigilance should maintain a strong focus on patients ‘actual experiences, concerns, and outcomes, and this approach can be expected to uncover hidden adverse event signals earlier and to help us understand adverse events in a patient-centred way. Patient-generated tōbyōkiblogs in the TOBYO database showed unique characteristics that were different from the data in existing sources generated by health care professionals. Analysis of tōbyōkiblogs would add value to the assessment of disorders with a high prevalence in women, psychiatric disorders in which subjective symptoms have important clinical meaning, refractory disorders, and other chronic disorders. | No | No |  |
| Matsuda 2017b[50] | Drug used in depression and rheumatoid arthritis | All | Not reported in enough detail. Abstract only | No | No | No | NR | NR | SRS | Frequency Type | Comparison of medical terms observed in t≈çby≈çki blogs with those in an external ADR reporting database showed that subjective and symptomatic events and general terms tended to be frequently observed in t≈çby≈çki blogs (e.g. anxiety, headache, and pain) while events using more technical medical terms (e.g. syndrome and abnormal laboratory test result) tended to be observed frequently in the ADR database. Exceptionally, the fact that ‚Interstitial lung disease‚Äù in patients with rheumatoid arthritis was observed frequently in both t≈çby≈çki blogs and the ADR database suggests relatively high attention for this event | It was suggested the TOBYO database may have the advantage of enabling analysis of patient-level outcomes that cannot be captured in existing data sources. | No | No |  |
| Natsiavas 2017[51] | NA (collected based on AE mentions) | Named - “acute liver injury”, “acute myocardial infarc‐tion”, “acute renal failure”, and “gastrointestinal bleeding” | Not reported in enough detail. | No | No | No | NR | 1954 | Online Medical Site | Novelty | Preliminary results show possible ADRs signals extracted from Twitter using their methods. Causality not established. No report of drugs associated with AE mentions. | Summarizing the presented platform’s overall contribution, it first facilitates the systematic real-time aggregation of free-text data sources with different characteristics. The standards-based semantic integration of the collected data could significantly facilitate further processing (e.g. knowledge engineering, analytics, etc.). These characteristics are very useful in public health surveillance applications, like the ADR signal detection scenario that we elaborated as a proof-of-concept. | No | No |  |
| Nguyen 2017[52] | alprazolam (Xanax), sertraline (Zoloft), citalopram (Celexa), fluoxetine (Prozac), lorazepam (Ativan), trazodone (Desyrel), escitalopram (Lexapro), duloxetine (Cymbalta), bupropion (Wellbutrin), venlafaxine (Effexor) | All | Lexical match did extend the lexicon using word2vec to improve coverage. No validation of AEs extracted (experienced, negated) | No | No | No | 602,799 posts | NR | SIDER | Frequency Correlation | It was found that across all drugs, in general, the rate of ADR derived using the extended lexicon is more accurate than using the original lexicon when compared with the SIDER rates. For example, for“diarrhoea”,the rate of documents in social media mentioned terms in the extended lexicon, across the drugs, is 0.78 correlated with the SIDER rate while that of the original lexicon is only 0.36. | The estimates were compared with the rates in SIDER database, an official source of drug side effects. The result indicated that the quantities derived from the proposed approach had better agreements with the official values. The work also demonstrates the efficiency of advanced computing framework in dealing with big data, providing prompt reports. | No | No |  |
| Ransohoff 2018[53] | erlotinib (Tarceva), nivolumab (Keytruda), pembrolizumab (Opdivo) | Named - Skin Adverse Effects | Not reported in enough detail. Extended abstract | No | No | No | 8 million posts | 3513 | Trials  Literature | Frequency Timing | We compared causal drug-ADR mentions of erythematous eruption and nail changes with erlotinib, and psoriasis flares and blistering reactions with immune check point inhibitors in the Inspire database with first-published clinical reports. Known ADRs were reported at frequencies comparable with those of published reports but with significantly enriched PRR scores and an average lead time of 7 months in advance of literature reporting4,5(range, 3-9 months). In addition, we detected 23 novel cases of hyperhidrosis in patients receiving erlotinib with an enriched PRR score of 1.90, which may represent a rare, missed ADR that has been present in online discussion for more than 11 years. | Our deep learning pipeline extracts mentions of cutaneous ADRs with high precision from the highly informal text in social health networks, detecting ADRs with an average 7-month lead-time from clinical reports. In addition, it uncovered a novel cutaneous ADR, not previously reported. | No | Yes |  |
| Nikfarjam 2019[54] | erlotinib, nivolumab and pembrolizumab | All |  | No | No | No | 50,574 : EGFR inhibitors and 16,598 checkpoint inhibitors | a total number of13,600 ADR concepts extracted from the former and 812 concepts from the latter | Literature | Frequency Timing Proportion | Known ADRs were associated with higher proportional reporting ratios compared to negative controls, demonstrating the robustness of our analyses. Our named entity recognition system achieved a 0.738 micro averaged F-measure in detecting ADR entities, not limited to cutaneous ADRs,in health forum posts. Additionally, we discovered the novel ADR of hypohidrosis reported by 23 patients in erlotinib-related posts; this ADR was absent from 15 years of literature on this medication, and we recently reported the finding in a clinical oncology journal. | We demonstrated the ability of a natural language processing-based signal-generation pipeline to accurately detect patient reports of ADRs months in advance of literature reporting and the robustness of statistical analyses to validate system detections. Our findings suggest the important contributions that social health network data can play in contributing to more comprehensive and timely pharmacovigilance. | No | No |  |
| Ransohoff 2018[55] | erlotinib, nivolumab and pembrolizumab | Named - chemotherapeutic skin toxicities | No | No | No | 8 million posts | NR | Literature | Frequency Timing  Type  Proportion |  | Our approach detected cutaneous ADRs with ahigh micro-average high precision (0.90). Known ADRs were reported at frequencies com-parable to published reports, but with an average lead-time of 7 months (range: 3-9 months) in advance of literature reporting and with significantly enriched PRRs. In addition, we detected 23 novel cases of hypohidrosis in patients receiving erlotinib, which may represent a rare, missed ADR that has been present in online discussion for over 11 years. | We demonstrate the capacity of deep learning-based methods to detect ADRs from online health forums, offering the potential for real-time pharmaco surveillance with rapid discovery of ADRs pre-ceding FDA detection and published clinical reports. | No | No |  |
| Oyebode 2023[56] | diabetes medications | All | 3rd Party: MetaMap | No | Yes | No | 6797 | 2572 (684 unique) | Sider Literature | Type | While a total of 313 ADRs were associated with Metformin in our data, SIDER captured 118 ADRs for Metformin. Some of SIDER’s ADRs are categories capturing other related ADRs (e.g., Gastrointestinal symptom NOS which captures other symptoms related to stomach and intestine, and Infection which captures ADRs that are infections). While most of our Metformin ADRs were found on SIDER overall, some were still missing. Majority of other Metformin’s ADRs such as nausea, abdominal bloating, vomiting, anorexia, headache, constipation, flatulence, asthenia, etc. are already captured in SIDER, but some ADRs were still missing in SIDER such as retrograde ejaculation, insomnia, pneumonia, gurgling, equilibration disorder, myocardial infarction, etc Furthermore, our results agree with (and extend) those reported by existing ADR research. For instance, in different studies conducted, patients placed on Metformin reported diarrhoea, nausea, anorexia or decreased appetite, vomiting, headache, tiredness or fatigue, abdominal pain, dizziness, constipation, stomach discomfort or upset stomach, hyperglycaemia as major ADRs. In addition, a research based on social media also reported diarrhoea, nausea, dizziness, and vomiting as top ADRs for Metformin. These findings support our results for Metformin and further corroborates the quality of our approach for detecting ADRs on a much larger scale. | Our findings showed that ADF is able to identify previously unknown ADRs after benchmarking with the SIDER database. | No | No |  |
| Park 2022[57] | tramadol and combination drugs | All | Manual extraction | No | Yes | No | NR | 869 | SRS Literature | Frequency Type Proportion | From the 869 AEs reported, we identified 125 new signals related to tramadol use not listed on the drug label that satisfied all 3 signal detection criteria. In addition, 20 serious AEs were selected from new signals. Among new serious AEs,vascular disorders had the largest signal detection criteria value. Based on the disproportionality analysis and patients’ symptom descriptions, tramadol-induced pain might also be an unexpected AE. | This study detected several novel signals related to tramadol use, suggesting newly identified possible AEs. Additionally, this study indicates that unexpected AEs can be detected using social media analysis alongside traditional pharmacovigilance data. | No | No |  |
| Pan 2018[58] | All | All | Not reported in enough detail | No | No | No | 450 symptom/AEs (breakdown of AEs only not specified) | 450 AE/symptom pairs | Literature | Frequency Type Proportion | Confirmed previously known findings from published studies and uncovered new and otherwise less frequently reported symptoms/AE | Supplementing traditional approaches through analysis of social media in this way can provide additional insights and help towards incorporating the patient perspective. | No | No |  |
| Patel 2018[59] | prednisolone or prednisone | All |  | No | No | No | 159,279 | 26,894 | SRS | Frequency | Insomnia and weight gain disproportionately reported in SM | Insomnia and weight gain are the most frequently reported glucocorticoid-related AEs posted on Twitter. There remains a disconnect between the frequency of these patient reported adverse events and our collective knowledge about these events. Here, using glucocorticoids as an example, we have demonstrated that Twitter can be a potentially useful, supplementary source for post marketing pharmacovigilance. Also found data on patient perspectives and quality of life. | No | No |  |
| Pathak 2023[60] | 35 drug-event pairs | Named - Joint pain; Stiffness; decrease in motion; skin redness; arthralgia; arthritis and joint swelling, Anaphylactic reaction; anaphylaxis; allergic reaction; hypersensitivity; hives; flushed skin; paleness; lump in throat; difficulty swallowing; sneezing; wheezing; tingling hands; swollen tongue; swollen lips |  | No | No | No | 61,661 | 1411 tweets were found to have one or more AEs (577 AEs for drug of interest) | SRS SPL/SPC | Frequency Proportion | Tweets for 15 out of the 35 drugs (42.9%) contained AEs associated with the signals. On pooling data from Twitter and Yellow Card, 24 out of 35 drug-event pairs (68.6%) were identified prior to the respective PRAC meetings. Both data sources showed similar distribution of AEs based on seriousness, however, the distribution based on labelling was divergent. | Twitter cannot be used in isolation for signal detection in current pharmacovigilance (PV) systems. However, it can be used in combination with traditional PV systems for early signal detection, as it can provide a holistic drug safety profile. | Yes | No |  |
| Pierce 2017[61] | lisdexamfetamine, olmesartan, methylphenidate, econazole, pradaxa, dronedarone, hydroxyzine, ziprasidone, tecfidera, sunscreen spray | Named - Raynaud’s phenomenon, Sprue-like enteropathy, Priapism, Increased international normalized ratio (INR) , Angioedema, Vasculitis , Serious injection site reactions, Drug reaction with eosinophilia and systemic symptoms (DRESS), Progressive multifocal leukoencephalopathy (PML), Flammability resulting in serious skin burns |  | Yes | No | No | 935,246 posts | 98,252 | SRS | Type  Timing | A total of 935,246 posts were harvested from Facebook and Twitter, from March 2009 through October2014. The automated classifier identified 98,252 Proto-AEs. Of these, 13 posts were selected for causality assessment of product–event pairs. Clinical assessment revealed that posts had sufficient information to warrant further investigation for two possible product–event association: dronedarone–vasculitis and Banana Boat Sun-screen–skin burns. No product–event associations were found among the negative controls. In one of the positive cases, the first report occurred in social media prior to signal detection from FAERS, whereas the other case occurred first in FAERS. | An efficient semi-automated approach to social media monitoring may provide earlier insights into certain adverse events. More work is needed to elaborate additional uses for social media data in pharmacovigilance and to determine how they can be applied by regulatory agencies. | No | No |  |
| Powell 2022[62] | denosumab, pembrolizuma | Named - multiple vertebral fractures following discontinuation (denosumab), immune-mediated skin adverse reactions (which includes Stevens–Johnson syndrome, toxic epidermal necrolysis, exfoliative dermatitis, and bullous pemphigoid)(pembrolizumab) | Lexical matching to UMLS or MedDRA | No | Yes | Yes | 36132 | 23764 (any AE): 316 AEOI | SRS | Frequency Timing | AEOI reported less frequently in SM and has. similar trend in increasing frequency as FAERS. Relevant medical insight needed to be extracted across multiple posts | our findings do not suggest that social media can highlight emerging issues earlier than spontaneous reports. Our results are therefore consistent with a recent publication by The Innovative Medicines Initiative WEB-RADR (Recognizing Adverse Drug Reactions) consortium, a partnership including members from European regulatory agencies, MAHs, academia and patient groups, who concluded that social media was not recommended for broad statistical signal detection. Linking posts longitudinally offers the ability to potentially generate more robust medical/AE insight and actively engaging patients online may enable rapid, more complete follow-up information for AEs. | Yes | No |  |
| Rees 2018[63] | 15 unnamed marketed products | All | 3rd party: Epidemico | Yes | No | No | NR | 947 (167 when compared to the core data sheet) | SRS | Type  Timing Proportion | Reported aEs are different on social media wrt type of event and seriousness and may contain more product complaints, or lack of benefits. No new safety signals were detected | The data that are currently available for pharmacovigilance are relatively limited (Twitter, some patient forums) due to access and privacy considerations. In addition, the unstructured nature of social media posts, together with use of informal and nonmedical language make the data that are available difficult to mine in a systematic way. With these caveats in mind it is perhaps not surprising that our pilot study, along with WEB-RADR and other research to date, do not provide sufficient evidence that signal detection using social media can detect signals not found in other datasets or earlier than traditional method. there may be some areas where information present in social media could be valuable to augment traditional data sources or in signal evaluation. Also found data on Patient Perspective; Quality of life; abuse. | No | No |  |
| Sadeghi 2017[64] | Direct Oral Anticoagulants (DOAs) | All | Not reported in enough detail. Abstract only - no details | No | No | No | NR | 235 | SRS | Frequency Type | More AEs labelled as “others” were found in websites (n = 205, 87.2%) including mainly arthralgia (n = 18, 8.8%), asthenia (n = 17, 8.3%) and headache (n = 14, 6.8%) compared to the FPVD (n = 2594, 26.7%) where ADRs labelled as “others” were mainly pruritus (n = 80, 2.8%), asthenia (n = 72, 2.5%) and acute renal failure (n = 63, 2.2%). Haemorrhagic effects were most frequently recorded in the FPVD (n = 4495, 56.8%) compared to websites (n = 28, 11.9%). AEs reported in websites were less “serious” (n = 16, 21%) than those recorded in FPVD (n = 3827, 80%) | There are differences between AEs reported in forums and ADRs reported by health professionals. As reported by other studies, data from patients’ websites and online discussions would complement those of FPVD and could be used as another source of data in pharmacovigilance to detect ADRs that could affect patients’ quality of life. Data on Quality of life. | No | No |  |
| Salamun 2020[65] | Tigecycline, aspirin | All | Lexical match of side effect to SIDER for extraction, unsure about statistical methods | No | No | No | 1023 | NR | SRS | Language | The results show the ability of the openFDA and social media sites to create real-time drug safety profiles by applying the same statistical methods applied in clinical trials. The social media data does not perform equally across all drug types and gives best results when applied to common over-the-counter drugs as opposed to last line of defence medications. | Adding other data sources would increase number of drug-ADE pair signals found | No | No |  |
| Sampathkumar 2017[66] | All | All | Extraction using lexical match - side effect dictionary created from SIDER, drug name dictionary using drugs.com. Automated annotations used, manually annotated 2000 For training/testing. Model train using only one forum. No validation of extracted data | No | No | No | 10,065 | 4670 (only high frequency drugs reported) | SPL/SPC Regulatory Agency Communications | Type  Timing | Many side effects extracted were similar to those in package inserts, however, there were some novel AEs (not on product labels) extracted from forums posts, including AEs later reported in safety communications/label updates | We have shown that the information extracted from this system matches published information available in Drug Package Inserts. In addition we have also been able to identify some novel adverse side-effect information that can act as early indicators for health authorities to help in their efforts towards Pharmacovigilance | No | No |  |
| Smith 2018 | adalimumab | All |  | No | No | No | 10,188 tweets | 801 | SRS Literature | Frequency Rank Proportion | There was moderate agreement between ADRs in social media and traditional sources. “Local and injection site reactions” was the top ADR in Twitter, DIDs, and systematic reviews by frequency, ranked frequency, and index ranking. The next highest ADR in Twitter—fatigue—ranked fifth and seventh in FAERS and DIDs. | Social media posts often express mild and symptomatic ADRs, but rates are measured differently in scientific sources. ADRs in FAERS are reported as absolute numbers, in DIDs as percentages, and in systematic reviews as percentages, risk ratios, or other metrics, which makes comparisons challenging; however, overlap is substantial. Social media analysis facilitates open-ended investigation of patient perspectives and may reveal concepts (e.g. anxiety) not available in traditional sources. Also found data on Patient Perspective. | No | No |  |
| Song 2021[67] | tofacitinib, baricitinib, and upadacitinib | All | Manual extraction | No | No | No | 130: tofacitinib (104), baricitinib (5), and upadacitinib (21) | 171 | SRS | Type | Twenty-two potential AEs were reported from FAERS and WebMD, and among 104 reviews in WebMD, fatigue (15) and weight increase (23) were the frequently reported potential AEs in patients taking tofacitinib. Although AEs such as nausea, dizziness, insomnia, constipation, and pruritus were reported from KAERS and WebMD, they were not included in the potential AEs due to disproportionality analysis using the FAERS database. | the present study suggested that the commonly reported potential AEs after the use of JAK inhibitors were increased risk of infection and thromboembolism. Unexpected AEs, such as malignancy and respiratory disorders, might also occur. However, there were some differences in the potential AEs frequently reported by JAK inhibitors. When an online patient review was integrated, ineffectiveness of the drug and gastrointestinal AEs were frequently reported for tofacitinib, infection was frequently reported with baricitinib, and symptoms related to pain or edema were mostly reported for upadacitinib. Since baricitinib and upadacitinib have been used for a relatively short period after marketing compared totofacitinib, further research using various real-world databases is necessary to find potential AEs related to JAK inhibitors inpatients with RA | No | No |  |
| Xia 2022[68] | all | All |  | No | No | No | 205746 | NR | Regulatory Agency Communications | Timing | The HAMLE framework detected more ADEs that previous methods and the detected new ADEs earlier than reported in safety comms | Our experiments confirmed that each level of embedding contributed to the performance improvement, and their combination achieved a new state-of-the-art result for ADE extraction | No | No |  |
| Yahya 2022[69] | gabapentin (Neurontin), lamotrigine (Lamictal ), topiramate (Topamax), pregabalin (Lyrica), clonazepam (Clonazepam), divalproex (SodiumDepakote), diazepam (Valium), oxcarbazepine (Trileptal), carbamazepine (Tegretol), levetiracetam (Keppra), phenytoin (Dilantin), acetazolamide (Diamox), duloxetine (Cymbalta), venlafaxine (Effexor), escitalopram (Lexapro), sertraline (Zoloft), bupropion (Wellbutrin), citalopram (Celexa), paroxetine (Paxil), pioglitazone (Actos), exenatide (Byetta), glucophage (Metformin), liraglutide (Victoza), lisinopril, carvedilol (Coreg), propranolol (Inderal), telmisartan (Micardis), cetirizine (Zyrtec), loratadine (Claritin), fexofenadine (Allegra), diphenhydramine (Benadryl), azelastine (Astelin), hydroxyzine (Vistaril), levocetirizine (Xyzal), omeprazole (Prilosec), esomeprazole (Nexium), lansoprazole (Prevacid), pantoprazole (Protonix), rabeprazole (Aciphex), ranitidine (Zantac), famotidin (Pepcid) | All | Found ADEs using lexical match (DIEGO LAB lexicon). Unsure of validation methods | No | No | No | 12,930 AED reviews/43,085 background drugs | 2093 | Sider Literature | Type | The analysis of the generated ADR lists indicates that most AED ADRs are of CNS type which is concordant with the extant pharmaceutical AED literature. | The consistency of the drawn answers with the existing pharmaceutical knowledge suggests the utility of the data from online health communities for AED-related knowledge discovery tasks. | Yes | No |  |
| Yahya 2022[70] | gabapentin (Neurontin), lamotrigine (Lamictal ), topiramate (Topamax), pregabalin (Lyrica), clonazepam (Clonazepam), divalproex (SodiumDepakote), diazepam (Valium), oxcarbazepine (Trileptal), carbamazepine (Tegretol), levetiracetam (Keppra), phenytoin (Dilantin), acetazolamide (Diamox), duloxetine (Cymbalta), venlafaxine (Effexor), escitalopram (Lexapro), sertraline (Zoloft), bupropion (Wellbutrin), citalopram (Celexa), paroxetine (Paxil), pioglitazone (Actos), exenatide (Byetta), glucophage (Metformin), liraglutide (Victoza), lisinopril, carvedilol (Coreg), propranolol (Inderal), telmisartan (Micardis), cetirizine (Zyrtec), loratadine (Claritin), fexofenadine (Allegra), diphenhydramine (Benadryl), azelastine (Astelin), hydroxyzine (Vistaril), levocetirizine (Xyzal), omeprazole (Prilosec), esomeprazole (Nexium), lansoprazole (Prevacid), pantoprazole (Protonix), rabeprazole (Aciphex), ranitidine (Zantac), famotidin (Pepcid) | All | Lexical matching only (Diego lab ADR lexicon). Not sure about the validation method | No | No | No | 12930 AED reviews/43085 background drugs | 2093 | SRS Sider Literature | Type | Less ADEs found in forum data than listed in SIDER, detected ADRs lists indicates that most AEDs ADRs are of CNS type which is concordant with the extant pharmaceutical AEDs literature | The qualitative and qualitative evaluation of the proposed methodology of detecting ARs of AEDs provide evidence on the validity of the proposed methodology and utility of the OCHs data for the knowledge discovery tasks related to AEDs. | No | No |  |
| Yu 2022[71] | 4888 drug names | All | Performance was low. They trained using pre-annotated dataset, no error analysis was done to explain the performance issues except it seems like their drug list was missing drug names in tweets. They used the output of their system for their qual analysis so the data reliability is questionable as their methods may have missed classified many tweets. No report on details of qual analysis (e.g., number of coders, codebook, agreement). Limited detail of ADE mentioned that were included in the themes | No | No | No | 34,586 | 941 tweets (640 ADEs for top 7) | Online Medical Site | Type | Found 70-78% of ADEs in tweets were known when compared to MedlinePlus with the rest newly reported in Twitter (please see my Note for comments on this) | Our validation study suggests that Twitter data not only include a sufficiently wide range of ADE mentions but also cover most known adverse reactions for drugs found in the relevant tweets. Our validation study suggests that Twitter data not only include a sufficiently wide range of ADE mentions but also cover most known adverse reactions for drugs found in the relevant tweets. | No | No |  |
| Zhou 2020[72] | hydrocodone/acetaminophen, levothyroxine, prednisone, lisinopril, amoxicillin, gabapentin, metformin, atorvastatin, alprazolam, and amlodipine | All | Manual review of tweets from advance search, not API, unsure if searched all at once, or one at a time. The % of tweets returned using this this method is not stated. No tweets collected means study is not reproducible. Method did not yield many results for some drugs, excluded 5 from analysis due to number of tweets being below threshold set. No report of number of coders or IAA if more than one | No | Yes | Yes | >40000 | NR | SRS | Frequency | Only looked at top 5 reported for each drug finding relative frequencies were not significantly different overall. | FAERS and Twitter shared similarities in types of data reported and a few unique items to each data set the use of Twitter as an ADR pharmacovigilance platform as well should continue to be studied as a unique and complementary source of information rather than a validation tool of existing ADR databases. | No | No |  |

References

1. Abbasi A, Li J, Adjeroh D, Abate M, Zheng W. Don’t mention it? Analyzing user-generated content signals for early adverse event warnings. Information Systems Research. 2019;30(3):1007-28. PMID: doi.org/10.1287/isre.2019.0847 doi: 10.2196/19266.

2. Karapetiantz P, Audeh B, Faille J, Lillo-Le Louet A, Bousquet C. Qualitative and Quantitative Analysis of Web Forums for Adverse Events Detection: "Strontium Ranelate" Case Study. Studies in health technology and informatics. 2019 21 Aug;264:964-8. PMID: 629152078. doi: <https://dx.doi.org/10.3233/SHTI190367>.

3. Bellet F, Lillo-Le Louet A, Karapetiantz P, Leprovost D, Grouin C, Bissan A, et al. Evaluation of online discussion forums as a complementary source of data for pharmacovigilance: the Vigi4MED project. Fundamental and Clinical Pharmacology. 2018;32(Supplement 1):45. PMID: 622650102. doi: <https://dx.doi.org/10.1111/fcp.12372>.

4. Karapetiantz P, Lillo-Le Louet A, Bousquet C. Informativity of French web forums for the evaluation of side effects of baclofen. Therapie. 2019 December;74(6):569-78. PMID: 2002165521. doi: <https://dx.doi.org/10.1016/j.therap.2019.05.003>.

5. Boeuf M, Bellet F, Karapetiantz P, Leprovost D, Morlane-Hondere F, Grouin C, et al. A pilot study of the Vigi4MED project: Comparison of adverse drug reactions (ADRs) of duloxetine between patients' forum posts and the French pharmacovigilance database (FPVD). Fundamental and Clinical Pharmacology. 2017;31(Supplement 1):33. PMID: 615222988.

6. Karapetiantz P, Audeh B, Lillo-Le Louët A, Bousquet. Signal Detection for Baclofen in Web Forums: A Preliminary Study. Studies in health technology and informatics. 2018 2018;247. PMID: 29677995.

7. Karapetiantz P, Bellet F, Audeh B, Lardon J, Leprovost D, Aboukhamis R, et al. Descriptions of Adverse Drug Reactions Are Less Informative in Forums Than in the French Pharmacovigilance Database but Provide More Unexpected Reactions. Frontiers in Pharmacology. 2018;9:439. PMID: 29765326. doi: <https://dx.doi.org/10.3389/fphar.2018.00439>.

8. Audeh B, Bellet F, Beyens MN, Lillo-Le Louet A, Bousquet C. Use of Social Media for Pharmacovigilance Activities: Key Findings and Recommendations from the Vigi4Med Project. Drug Saf. 2020;43(9):835-51. PMID: 32557179. doi: <https://dx.doi.org/10.1007/s40264-020-00951-2>.

9. Barakat NH, ElSabbagh AH. From Similarities to Probabilities: Feature Engineering for Predicting Drugs' Adverse Reactions. Intell Autom Soft Comput. 2022;32(2):1207-24. PMID: WOS:000720844200011. doi: 10.32604/iasc.2022.022104.

10. Bennett CL, Gundabolu K, Kwak LW, Djulbegovic B, Champigneulle O, Josephson B, et al. Using Twitter for the identification of COVID-19 vaccine-associated haematological adverse events. The Lancet Haematology. 2022 January;9(1):e12-e3. PMID: 2016043533. doi: <https://dx.doi.org/10.1016/S2352-3026%2821%2900378-1>.

11. Bhattacharya M, Snyder S, Malin M, Truffa MM, Marinic S, Engelmann R, et al. Using Social Media Data in Routine Pharmacovigilance: A Pilot Study to Identify Safety Signals and Patient Perspectives. Pharmaceutical Medicine. 2017 01 Jun;31(3):167-74. PMID: 616410571. doi: <https://dx.doi.org/10.1007/s40290-017-0186-6>.

12. Blaser DA, Eaneff S, Loudon-Griffiths J, Roberts S, Phan P, Wicks P, et al. Comparison of rates of nausea side effects for prescription medications from an online patient community versus medication labels: an exploratory analysis. AAPS Open. 2017;3(1) (no pagination). PMID: 619429002. doi: <https://dx.doi.org/10.1186/s41120-017-0020-y>.

13. Borchert JS, Wang B, Ramzanali M, Stein AB, Malaiyandi LM, Dineley KE. Adverse Events Due to Insomnia Drugs Reported in a Regulatory Database and Online Patient Reviews: Comparative Study. J Med Internet Res. 2019;21(11):14. PMID: WOS:000495465800001. doi: 10.2196/13371.

14. Brattig Correia R. Prediction of Drug Interaction and Adverse Reactions, with Data from Electronic Health Records, Clinical Reporting, Scientific Literature, and Social Media, Using Complexity Science Methods. United States -- Indiana: Indiana University; 2019.

15. Campillos-Llanos L, Grouin C, Lillo-Le Louet A, Zweigenbaum P. Initial Experiments for Pharmacovigilance Analysis in Social Media Using Summaries of Product Characteristics. Stud Health Technol Inform. 2019 Aug 21;264:60-4. PMID: 31437885. doi: <https://dx.doi.org/10.3233/SHTI190183>.

16. van Stekelenborg J, Ellenius J, Maskell S, Bergvall T, Caster O, Dasgupta N, et al. Recommendations for the Use of Social Media in Pharmacovigilance: Lessons from IMI WEB-RADR. Drug Safety. 2019 01 Dec;42(12):1393-407. PMID: 2002629493. doi: <https://dx.doi.org/10.1007/s40264-019-00858-7>.

17. Caster O, Dietrich J, Kurzinger ML, Lerch M, Maskell S, Noren GN, et al. Assessment of the Utility of Social Media for Broad-Ranging Statistical Signal Detection in Pharmacovigilance: Results from the WEB-RADR Project. Drug Safety. 2018 12;41(12):1355-69. PMID: 30043385. doi: <https://dx.doi.org/10.1007/s40264-018-0699-2>.

18. Chen X, Faviez C, Schuck S, Lillo-Le-Louet A, Texier N, Dahamna B, et al. Mining Patients' Narratives in Social Media for Pharmacovigilance: Adverse Effects and Misuse of Methylphenidate. Frontiers in Pharmacology. 2018;9:541. PMID: 29881351. doi: <https://dx.doi.org/10.3389/fphar.2018.00541>.

19. De Langen J, Lahary JC, Gouraud A, Vial T, Le Priol Y. Contribution of social media content monitoring to the identification of suspected adverse reactions to birth control arm implants: A comparison with literature monitoring. Fundamental and Clinical Pharmacology. 2017 April;31(Supplement 1):15. PMID: 615223372.

20. den Hollander D, Dirkson AR, Verberne S, Kraaij W, van Oortmerssen G, Gelderblom H, et al. Symptoms reported by gastrointestinal stromal tumour (GIST) patients on imatinib treatment: combining questionnaire and forum data. Support Care Cancer. 2022;30(6):5137-46. PMID: 35233640. doi: <https://dx.doi.org/10.1007/s00520-022-06929-3>.

21. Dirkson A, Verberne S, Kraaij W, van Oortmerssen G, Gelderblom H. Automated gathering of real-world data from online patient forums can complement pharmacovigilance for rare cancers. Scientific Reports. 2022 06 20;12(1):10317. PMID: 35725736. doi: <https://dx.doi.org/10.1038/s41598-022-13894-8>.

22. De Rosa M, Fenza G, Gallo A, Gallo M, Loia V. Pharmacovigilance in the era of social media: Discovering adverse drug events cross-relating Twitter and PubMed. Futur Gener Comp Syst. 2021;114:394-402. PMID: WOS:000579186700034. doi: 10.1016/j.future.2020.08.020.

23. Dreyfus B, Pierce CE. Social media compared to faers and administrative claims for pharmacovigilance. Pharmacoepidemiology and Drug Safety. 2017 August;26(Supplement 2):512-3. PMID: 618126008. doi: <https://dx.doi.org/10.1002/pds.4275>.

24. Eslami B, Rezaei Z, Habibzadeh M, Fouladian M, Ebrahimpour-Komleh H. Using deep learning methods for discovering associations between drugs and side effects based on topic modeling in social network. Soc Netw Anal Min. 2020;10(1):17. PMID: WOS:000535336500001. doi: 10.1007/s13278-020-00645-8.

25. Farooq H, Niaz JS, Fakhar S, Naveed H. Leveraging digital media data for pharmacovigilance. AMIA Annu Symp Proc. 2020;2020:442-51. PMID: 33936417.

26. Ferawati K, Liew K, Aramaki E, Wakamiya. Monitoring Mentions of COVID-19 Vaccine Side Effects on Japanese and Indonesian Twitter: Infodemiological Study. JMIR infodemiology. 2022 10/04/2022;2(2). PMID: 36277140. doi: 10.2196/39504.

27. Gavrielov-Yusim N, Kurzinger ML, Nishikawa C, Pan C, Pouget J, Epstein LB, et al. Comparison of text processing methods in social media-based signal detection. Pharmacoepidemiol Drug Saf. 2019;28(10):1309-17. PMID: 31392844. doi: <https://dx.doi.org/10.1002/pds.4857>.

28. Golder S, Smith K, O'Connor K, Gross R, Hennessy S, Gonzalez-Hernandez G. A Comparative View of Reported Adverse Effects of Statins in Social Media, Regulatory Data, Drug Information Databases and Systematic Reviews. Drug Saf. 2021;44(2):167-79. PMID: 33001380. doi: <https://dx.doi.org/10.1007/s40264-020-00998-1>.

29. Han N, Oh JM, Kim IW. Assessment of adverse events related to anti-influenza neuraminidase inhibitors using the FDA adverse event reporting system and online patient reviews. Sci. 2020;10(1):3116. PMID: 32080337. doi: <https://dx.doi.org/10.1038/s41598-020-60068-5>.

30. Harpster E, Hultgren K. Ciprofloxacin and levofloxacin: Twitter versus food and drug administration adverse event reporting system. Journal of the American Pharmacists Association. 2018 May - June;58(3):e162. PMID: 622478660. doi: <https://dx.doi.org/10.1016/j.japh.2018.04.004>.

31. Hoang T, Liu J, Pratt N, Zheng VW, Chang KC, Roughead E, et al. Authenticity and credibility aware detection of adverse drug events from social media. Int J Med Inf. 2018;120:157-71. PMID: 30409341. doi: <https://dx.doi.org/10.1016/j.ijmedinf.2018.10.003>.

32. Hussain Z, Sheikh Z, Tahir A, Dashtipour K, Gogate M, Sheikh A, et al. Artificial Intelligence-Enabled Social Media Analysis for Pharmacovigilance of COVID-19 Vaccinations in the United Kingdom: Observational Study. JMIR Public Health and Surveillance. 2022 05 27;8(5):e32543. PMID: 35144240. doi: <https://dx.doi.org/10.2196/32543>.

33. Jarynowski A, Semenov A, Kaminski M, Belik V. Mild Adverse Events of Sputnik V Vaccine in Russia: Social Media Content Analysis of Telegram via Deep Learning. Journal of Medical Internet Research. 2021 11 29;23(11):e30529. PMID: 34662291. doi: <https://dx.doi.org/10.2196/30529>.

34. Jiang KY, Huang LY, Chen TY, Karbaschi G, Zhang DK, Bernard GR, editors. Mining Potentially Unreported Effects from Twitter Posts through Relational Similarity: A Case for Opioids. 2020; Electr Network: Ieee Computer Soc.

35. Khademi Habibabadi S, Palmer C, Dimaguila GL, Javed M, Clothier HJ, Buttery J. Australasian Institute of Digital Health Summit 2022-Automated Social Media Surveillance for Detection of Vaccine Safety Signals: A Validation Study. Appl Clin Inform. 2023;14(1):1-10. PMID: 36351547. doi: <https://dx.doi.org/10.1055/a-1975-4061>.

36. Kim H, Liang OS, Yang CC, Ieee Comp SOC, editors. Detecting Potential Adverse Drug Reactions of Preschool ADHD Treatment Using Health Consumer-Generated Content. 2020; Electr Network: Ieee Computer Soc.

37. Koutkias VG, Lillo-Le Louet A, Jaulent MC. Exploiting heterogeneous publicly available data sources for drug safety surveillance: computational framework and case studies. Expert Opin Drug Saf. 2017;16(2):113-24. PMID: 27813420. doi: <https://dx.doi.org/10.1080/14740338.2017.1257604>.

38. Kurzinger ML, Schuck S, Texier N, Abdellaoui R, Faviez C, Pouget J, et al. Web-Based Signal Detection Using Medical Forums Data in France: Comparative Analysis. J Med Internet Res. 2018;20(11):e10466. PMID: 30459145. doi: <https://dx.doi.org/10.2196/10466>.

39. Kurzinger ML, Gavrielov-Yusim N, Nishikawa C, Pan C, Pouget J, Epstein L, et al. Web-based signal detect ION using medical forums world wide from 2005 to 2015. Pharmacoepidemiology and Drug Safety. 2018;27(Supplement 2):261. PMID: 623864590. doi: <https://dx.doi.org/10.1002/pds.4629>.

40. Lardon J, Bellet F, Aboukhamis R, Asfari H, Souvignet J, Jaulent MC, et al. Evaluating Twitter as a complementary data source for pharmacovigilance. Expert Opin Drug Saf. 2018;17(8):763-74. PMID: 29991282. doi: <https://dx.doi.org/10.1080/14740338.2018.1499724>.

41. Lebanova H, Grigorov E, Tonev K. Online Discussion Forums in Bulgaria as a Source of Adverse Drug Reactions Reports for Omeprazole and Famotidine. Drug Saf. 2019;42(10):1219-20. PMID: WOS:000485893700034.

42. Lee S, Woo H, Lee CC, Kim G, Kim J-Y, Lee S. Drug_SNSMiner: standard pharmacovigilance pipeline for detection of adverse drug reaction using SNS data. Scientific reports. 2023;13(1):3779-NA. doi: 10.1038/s41598-023-28912-6.

43. Li S, Yu C-H, Wang Y, Babu Y. Exploring adverse drug reactions of diabetes medicine using social media analytics and interactive visualizations. International Journal of Information Management. 2019 Oct;48:228-37. PMID: 2019-50179-022. doi: <https://dx.doi.org/10.1016/j.ijinfomgt.2018.12.007>.

44. Li Y, Jimeno Yepes A, Xiao C. Combining Social Media and FDA Adverse Event Reporting System to Detect Adverse Drug Reactions. Drug Safety. 2020 01 Sep;43(9):893-903. PMID: 2004891656. doi: <https://dx.doi.org/10.1007/s40264-020-00943-2>.

45. Lian A, Du J, Tang L. Using a Machine Learning Approach to Monitor COVID-19 Vaccine Adverse Events (VAE) from Twitter Data. Vaccines. 2022 January;10(1) (no pagination). PMID: 2015288759. doi: <https://dx.doi.org/10.3390/vaccines10010103>.

46. Liu X. Health data analytics: Data and text mining approaches for pharmacovigilance. Dissertation Abstracts International Section A: Humanities and Social Sciences. 2017;77(12-A(E)):No Pagination Specified. PMID: 2016-58397-158.

47. MacKinlay A, Aamer H, Yepes AJ. Detection of Adverse Drug Reactions using Medical Named Entities on Twitter. AMIA Annual Symposium Proceedings/AMIA Symposium. 2017;2017:1215-24. PMID: 29854190.

48. Maskell S. When does social media add value to pharmacovigilance? Drug Safety. 2017 October;40(10):1040. PMID: 618778404. doi: <https://dx.doi.org/10.1007/s40264-017-0580-8>.

49. Matsuda S, Aoki K, Tomizawa S, Sone M, Tanaka R, Kuriki H, et al. Analysis of Patient Narratives in Disease Blogs on the Internet: An Exploratory Study of Social Pharmacovigilance. JMIR Public Health Surveill. 2017;3(1):e10. PMID: 28235749. doi: <https://dx.doi.org/10.2196/publichealth.6872>.

50. Matsuda S, Aoki K, Tomizawa S, Sone M, Tanaka R, Kuriki H, et al. Mining events appearing in patient narratives in disease blogs on the internet: Social pharmacovigilance. Pharmacoepidemiology and Drug Safety. 2017;26(Supplement 2):513. PMID: 618126020. doi: <https://dx.doi.org/10.1002/pds.4275>.

51. Natsiavas P, Maglaveras N, Koutkias V. KR4HC/ProHealth@HEC - A Public Health Surveillance Platform Exploiting Free-Text Sources via Natural Language Processing and Linked Data: Application in Adverse Drug Reaction Signal Detection Using PubMed and Twitter2017. 51-67 p.

52. Nguyen T, Larsen ME, O'Dea B, Phung D, Venkatesh S, Christensen H. Estimation of the prevalence of adverse drug reactions from social media. Int J Med Inf. 2017;102:130-7. PMID: 28495341. doi: <https://dx.doi.org/10.1016/j.ijmedinf.2017.03.013>.

53. Ransohoff JD, Nikfarjam A, Kwong B, Shah N, Sarin KY. Early detection of chemotherapeutic skin toxicities in social health networks using deep learning. Journal of Investigative Dermatology. 2018;138(5 Supplement 1):S42. PMID: 622253200.

54. Nikfarjam A, Ransohoff JD, Callahan A, Jones E, Loew B, Kwong BY, et al. Early Detection of Adverse Drug Reactions in Social Health Networks: A Natural Language Processing Pipeline for Signal Detection. JMIR Public Health Surveill. 2019;5(2):e11264. PMID: 31162134. doi: <https://dx.doi.org/10.2196/11264>.

55. Ransohoff JD, Nikfarjam A, Jones E, Loew B, Kwong BY, Sarin KY, et al. Detecting Chemotherapeutic Skin Adverse Reactions in Social Health Networks Using Deep Learning. JAMA oncology. 2018;4(4):581-3. doi: 10.1001/jamaoncol.2017.5688.

56. Oyebode O, Orji R. Identifying adverse drug reactions from patient reviews on social media using natural language processing. Health Informatics J. 2023 Jan-Mar;29(1):14604582221136712. PMID: 36857033. doi: 10.1177/14604582221136712.

57. Park S, Choi SH, Song YK, Kwon JW. Comparison of Online Patient Reviews and National Pharmacovigilance Data for Tramadol-Related Adverse Events: Comparative Observational Study. JMIR Public Health Surveill. 2022;8(1):e33311. PMID: 34982723. doi: <https://dx.doi.org/10.2196/33311>.

58. Pan S, Halhol S, Booth A, Cox A, Merinopoulou E. Profiling of Disease Symptoms and Adverse Events: Does Social Media Augment Traditional Approaches? Value in Health. 2018 October;21(Supplement 3):S356. doi: <https://dx.doi.org/10.1016/j.jval.2018.09.2130>.

59. Patel R, Belousov M, Jani M, Dasgupta N, Winokur C, Nenadic G, et al. Frequent discussion of insomnia and weight gain with glucocorticoid therapy: An analysis of Twitter posts. npj digit. 2018;1:12. PMID: 30740536. doi: <https://dx.doi.org/10.1038/s41746-017-0007-z>.

60. Pathak R, Catalan-Matamoros D. Can Twitter posts serve as early indicators for potential safety signals? A retrospective analysis. Int. 2023;34(1):41-61. PMID: 35491804. doi: <https://dx.doi.org/10.3233/JRS-210024>.

61. Pierce CE, Bouri K, Pamer C, Proestel S, Rodriguez HW, Van Le H, et al. Evaluation of Facebook and Twitter Monitoring to Detect Safety Signals for Medical Products: An Analysis of Recent FDA Safety Alerts. Drug Saf. 2017;40(4):317-31. PMID: 28044249. doi: <https://dx.doi.org/10.1007/s40264-016-0491-0>.

62. Powell G, Kara V, Painter JL, Schifano L, Merico E, Bate A. Engaging Patients via Online Healthcare Fora: Three Pharmacovigilance Use Cases. Front Pharmacol. 2022;13:901355. PMID: 35721140. doi: <https://dx.doi.org/10.3389/fphar.2022.901355>.

63. Rees S, Mian S, Grabowski N. Using social media in safety signal management: is it reliable? Ther. 2018;9(10):591-9. PMID: 30283627. doi: <https://dx.doi.org/10.1177/2042098618789596>.

64. Sadeghi S, Chebane L, Montastruc JL, Bagheri H. Adverse drug reactions related to direct oral anticoagulant: Patient's internet narratives versus pharmacovigilance database. Drug Safety. 2017;40(10):953. PMID: 618778599. doi: <https://dx.doi.org/10.1007/s40264-017-0580-8>.

65. Salamun A, Duque S, Madiraju P, editors. Analyzing Adverse Event Signal Detection with Publicly Available Web Sources. 2020; Electr Network: Ieee.

66. Sampathkumar H. A framework for information retrieval and knowledge discovery from online healthcare forums. Dissertation Abstracts International: Section B: The Sciences and Engineering. 2017;77(7-B(E)):No Pagination Specified. PMID: 2016-47711-148.

67. Song YK, Song J, Kim K, Kwon JW. Potential Adverse Events Reported With the Janus Kinase Inhibitors Approved for the Treatment of Rheumatoid Arthritis Using Spontaneous Reports and Online Patient Reviews. Front Pharmacol. 2021;12:792877. PMID: 35087406. doi: <https://dx.doi.org/10.3389/fphar.2021.792877>.

68. Xia L. Historical profile will tell? A deep learning-based multi-level embedding framework for adverse drug event detection and extraction. Decis Support Syst. 2022;160:12. PMID: WOS:000831691400001. doi: 10.1016/j.dss.2022.113832.

69. Yahya AA, Asiri Y, Alyami I. Social Media Analytics for Pharmacovigilance of Antiepileptic Drugs. Computational and Mathematical Methods in Medicine. 2022;2022 (no pagination). PMID: 2016534815. doi: <https://dx.doi.org/10.1155/2022/8965280>.

70. Yahya A, Asiri Y. Automatic Detection of Adverse Drug Reactions from Online Health Forums. 13th International Conference on Information and Communication Systems: IEEE; 2022.

71. Yu D, Vydiswaran VGV. An Assessment of Mentions of Adverse Drug Events on Social Media With Natural Language Processing: Model Development and Analysis. JMIR Med Inform. 2022 Sep 28;10(9):e38140. PMID: 36170004. doi: <https://dx.doi.org/10.2196/38140>.

72. Zhou Z, Hultgren KE. Complementing the US Food and Drug Administration Adverse Event Reporting System With Adverse Drug Reaction Reporting From Social Media: Comparative Analysis. JMIR public health and surveillance. 2020 30 Sep;6(3):e19266. PMID: 635735927. doi: <https://dx.doi.org/10.2196/19266>.
